# Global metabolomic profiling of tumor tissue and paired serum samples to identify biomarkers for response to neoadjuvant FOLFIRINOX treatment of human pancreatic cancer

**DOI:** 10.1002/1878-0261.13759

**Published:** 2024-11-15

**Authors:** Manoj Amrutkar, Sander Johannes Thorbjørnsen Guttorm, Anette Vefferstad Finstadsveen, Knut Jørgen Labori, Lars Eide, Helge Rootwelt, Katja Benedikte Prestø Elgstøen, Ivar P. Gladhaug, Caroline S. Verbeke

**Affiliations:** ^1^ Department of Pathology, Division of Laboratory Medicine Oslo University Hospital Norway; ^2^ Department of Medical Biochemistry, Division of Laboratory Medicine Oslo University Hospital Norway; ^3^ Core Facility for Global Metabolomics and Lipidomics, Institute of Clinical Medicine, Faculty of Medicine University of Oslo Norway; ^4^ Institute of Clinical Medicine, Faculty of Medicine University of Oslo Norway; ^5^ Department of Hepato‐Pancreato‐Biliary Surgery Oslo University Hospital Oslo Norway; ^6^ Department of Medical Biochemistry, Institute of Clinical Medicine, Faculty of Medicine University of Oslo Norway

**Keywords:** FOLFIRINOX, LC–MS, neoadjuvant chemotherapy, pancreatic cancer, untargeted metabolomics

## Abstract

Neoadjuvant chemotherapy (NAT) is increasingly used for the treatment of non‐metastatic pancreatic ductal adenocarcinoma (PDAC) and is established as a standard of care for borderline resectable and locally advanced PDAC. However, full exploitation of its clinical benefits is limited by the lack of biomarkers that assess treatment response. To address this unmet need, global metabolomic profiling was performed on tumor tissue and paired serum samples from patients with treatment‐naïve (TN; *n* = 18) and neoadjuvant leucovorin calcium (folinic acid), fluorouracil, irinotecan hydrochloride and oxaliplatin (FOLFIRINOX)‐treated (NAT; *n* = 17) PDAC using liquid chromatography mass spectrometry. Differentially abundant metabolites (DAMs) in TN versus NAT groups were identified and their correlation with various clinical parameters was assessed. Metabolomics profiling identified 40 tissue and five serum DAMs in TN versus NAT PDAC. In general, DAMs associated with amino acid and nucleotide metabolism were lower in NAT compared to TN. Four DAMs—3‐hydroxybutyric acid (BHB), 3‐carboxy‐4‐methyl‐5‐propyl‐2‐furanpropanoic acid (CMPF), glycochenodeoxycholate and citrulline—were common to both tissue and serum and showed a similar pattern of differential abundance in both groups. A strong positive correlation was observed between serum carbohydrate 19‐9 antigen (CA 19‐9) and tissue carnitines (C12, C18, C18:2) and N8‐acetylspermidine. The reduction in CA 19‐9 following NAT correlated negatively with serum deoxycholate levels, and the latter correlated positively with survival. This study revealed neoadjuvant‐chemotherapy‐induced changes in metabolic pathways in PDAC, mainly amino acid and nucleotide metabolism, and these correlated with reduced CA 19‐9 following neoadjuvant FOLFIRINOX treatment.

## Graphical abstract

Impact of neoadjuvant FOLFIRINOX on human PDAC was investigated using global metabolomic profiling of tumor tissue and serum samples of treatment‐naïve (TN) and neoadjuvantly treated (NAT) tumors. Of 45 metabolites with differential abundance in NAT versus TN, four were common to tissue and serum—BHB, CMPF, GCDC, and citrulline. Moreover, amino acid and nucleotide metabolic pathways were altered following NAT.

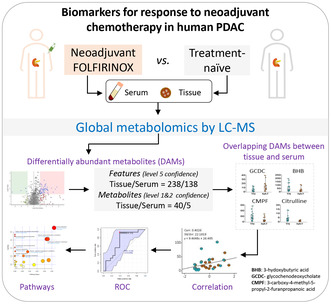

**Abbreviations**
AUC: area under the curve
BHB: β‐hydroxybutyric acid
CA 19‐9: carbohydrate 19‐9 antigen
CAP: College of American Pathologists
CMPF: 3‐carboxy‐4‐methyl‐5‐propyl‐2‐furanpropanoic acid
CRP: C‐reactive protein
DAM: differentially abundant metabolite
FOLFIRINOX: 5‐fluorouracil, leucovorin, irinotecan, and oxaliplatin
GCDC: glycochenodeoxycholate
LC–MS: liquid chromatography mass spectrometry
N8AS: N8‐acetylspermidine
NAT: neoadjuvant chemotherapy treated
PCA: principal component analysis
PDAC: pancreatic ductal adenocarcinoma
PQC: pooled quality control
ROC: receiver operating characteristic
TN: treatment‐naïve

## 1 Introduction

Pancreatic ductal adenocarcinoma (PDAC; pancreatic cancer) has a poor survival (5‐year survival of < 10%) and is expected to become the second leading cause of cancer‐related death in the Western world [1, 2]. Surgery is currently the only potentially curative treatment; however, most patients (> 80%) are inoperable at the time of diagnosis due to locally advanced or metastatic disease. Regardless of surgical eligibility, the clinical care of most PDAC patients includes systemic chemotherapy [3]. Gemcitabine monotherapy has been the standard chemotherapy for PDAC until the emergence of two combination chemotherapy regimens—FOLFIRINOX (5‐fluorouracil, leucovorin, irinotecan, and oxaliplatin) and gemcitabine/nab‐paclitaxel (Abraxane; gemcitabine bound to an albumin nanoparticle conjugate of paclitaxel) [4, 5]. Both combination regimens have become a first‐line treatment option for patients with advanced PDAC and a preferred choice for neoadjuvant treatment (NAT) [6].

Patients with resectable PDAC are given adjuvant chemotherapy to extend disease‐free and overall survival (OS); however, about 40% patients fail to receive adjuvant therapy due to surgical complications, early disease progression or poor performance status [7]. A multimodal approach consisting of neoadjuvant chemotherapy followed by surgery and adjuvant chemotherapy has been considered a possible treatment option for all non‐metastatic PDACs [8]. NAT has become a standard of care for borderline and locally advanced PDAC owing to its potential benefits to reduce tumor burden, control metastasis, and improve the selection of patients eligible for surgical resection [8, 9, 10, 11]. Despite these advantages, the benefits of NAT in resectable PDAC remain debatable [12, 13, 14, 15, 16]. Moreover, the rapid development of chemotherapy resistance, a hallmark of PDAC, continues to be a potential cause of concern when using NAT [17]. Hence, a detailed understanding of chemotherapy‐induced changes in the tumor and robust data about the actual clinical benefits of NAT are necessary for further improvement of PDAC treatment strategies.

Response to NAT in PDAC is currently evaluated preoperatively based on imaging and changes in serum CA 19‐9, and post‐operatively by assessing histological evidence of tumor regression [8, 9]. However, these methods have limited sensitivity and specificity, and their association with patient outcome is suboptimal. Moreover, the availability of pretreatment tumor samples is limited to an exceedingly small quantity of cells and tissue from diagnostic procedures. Therefore, comparison between treatment‐naïve (TN) and NAT‐treated surgically resected PDAC specimens is generally used as a proxy for assessment of the changes in PDAC before and after NAT [18, 19]. Relatively little is known about the molecular changes induced by NAT in PDAC and their potential association with patient outcome. Our recent study of proteome alterations in PDAC tissue in response to NAT has, for the first time, revealed altered metabolic profiles both at the tumor and systemic level [18]. Furthermore, a recent study reported a shift in the proteome profiles of extracellular matrix composition, complement activation, energy metabolism, and ribosomal proteins in the residual tumor mass of NAT compared to TN PDACs. Moreover, the observed shift was influenced by the chemotherapy regimen that had been used [19]. These studies highlight the importance of further investigations into NAT‐induced molecular changes in PDAC, particularly to identify specific biomarkers of treatment response.

The search for altered metabolites in tumor and serum samples from PDAC patients receiving NAT compared to patients undergoing upfront surgery seems an obvious way forward. Indeed, it is likely that the unique metabolites present in the biological samples mirror changes introduced by NAT more closely than alterations at the level of the genome, transcriptome, or proteome. Metabolomics is an advanced technique to profile small molecule metabolites (< 1.5 kDa), which are present in biofluids, cells, and tissues, and may provide crucial information about underlying pathophysiology. As such, metabolomics has become a useful tool for biomarker discovery for various diseases, including cancer, with respect to diagnosis, treatment selection, and monitoring of treatment response and disease progression [20, 21, 22]. The rapid biochemical response to pharmaceutical intervention may allow a much earlier evaluation of treatment response than assessment based on imaging, histology or clinical parameters.

In recent years, research on metabolomics in PDAC has focused on the discovery of biomarkers for early detection and prediction of clinical outcome [19, 23, 24, 25, 26, 27, 28, 29]. In this study, metabolomic profiling of tumor tissue and matched serum samples from PDAC patients who underwent surgical resection either upfront (TN) or following NAT with FOLFIRINOX was performed using untargeted ultra‐high‐performance liquid chromatography mass spectrometry (LC–MS). The main aim of the study was to characterize chemotherapy‐induced metabolic alterations in PDAC, with the purpose of identifying potential biomarkers for treatment response.

## 2 Materials and methods

### 2.1 Chemicals

Water (type 1; > 18 MΩ cm) was obtained from MilliQ ultrapure water purification system (Merck Millipore, Darmstadt, Germany). Methanol was purchased from Rathburn Chemicals (Walkerburn, UK) and Formic acid (LCMS grade) was from Thermo Scientific Chemicals (Waltham, MA, USA). Methyl Tert Butyl Ether (MTBE) was purchased from Sigma‐Aldrich (Steinheim, Germany). 2‐Propanol (IPA) was purchased from VWR Chemicals (Bergen, Norway).

### 2.2 Patients and samples

The study series consisted of tumor tissues sampled from surgical specimens and matched serum samples obtained from 35 PDAC patients who underwent surgical resection at Oslo University Hospital, Rikshospitalet, Oslo, Norway, in the years 2015–2022. Of these, 18 received upfront surgery (TN group) and 17 were treated with neoadjuvant FOLFIRINOX (NAT group). Clinicopathological information is provided in Table 1 and Table S1. Survival data were last updated in December 2023. Tissue sampling was carried out as described previously [18]. Briefly, sample collection and snap‐freezing were performed within ~ 20 min of surgical resection. Blood samples were collected on the day before surgery and were processed for serum extraction following routine standard procedures established at the hospital. All samples were stored at −80 °C until further use. For quality control purposes, tissue and serum samples from two patients with pancreatic disease other than PDAC (neuroendocrine tumor, autoimmune pancreatitis) were processed and included in the pooled quality control (PQC) samples described below in Section 2.4.

**Table 1:** Clinical characteristics of the study population. Age, BMI, tumor size, serum parameters, and survival data are presented with median values and min–max range, while the data for all other categories is presented in actual numbers and percentage. Overall survival was calculated from date of diagnosis. BMI, body‐mass index; CA 19‐9, carbohydrate 19‐9 antigen; CAP, College of American Pathologists; DP, distal pancreatectomy; PPPD, pylorus‐preserving pancreatoduodenectomy; TNM, tumor‐node‐metastasis; TP, total pancreatectomy.

Category	Treatment‐naïve (TN, *n* = 18)	Neoadjuvantly treated (NAT, *n* = 17)
Gender		
Male	13 (72%)	7 (41%)
Female	5 (28%)	10 (59%)
Age (years)	74.5 (50.0–84.0)	64.0 (51.0–75.0)*
BMI (kg·m^−2^)	23.8 (20.7–39.6)	24.7 (17.3–34.8)
Comorbidity (all)	15/18 (83%)	8/17 (47%)
Diabetes	8	2
Cardiovascular	8	2
Hypertension	5	3
Others	7	5
Disease stage		
Primary resectable	18 (100%)	6 (35%)
Borderline resectable	0	10 (59%)
Locally advanced	0	1 (6%)
Resection type		
PPPD	14 (78%)	16 (94%)
TP	0	1 (6%)
DP	4 (22%)	0 (0%)
Tumor size (mm)	37.0 (28.0–102.0)	35.0 (22.0–54.0)
TNM classification (8th edition)		
Tumor (T)		
T1	0	0
T2	5 (28%)	7 (41%)
T3	13 (72%)	10 (59%)
T4	0	0
Lymph node metastasis (N)		
N0	4 (22%)	2 (9%)
N1	5 (28%)	9 (41%)
N2	9 (50%)	11 (50%)
Tumor regression grade		
CAP 0	–	0
CAP 1	–	0
CAP 2	–	9 (53%)
CAP 3	–	8 (47%)
Serum parameters		
CA 19‐9 preoperative (U·mL^−1^)	290.5 (11.0–11371.0)	56.0 (7.0–477.0)
Bilirubin (μmol·L^−1^)	30.0 (5.0–341.0)	5.0 (3.0–24.0)*
Albumin (mg·dL^−1^)	40.5 (34.0–45.0)	42.0 (24.0–44.0)
C‐reactive protein (mg·L^−1^)	4.3 (0.6–30.0)	2.4 (0.8–17.0)^#^
Adjuvant chemotherapy	9/18 (50%)	16/17 (94%)
Overall survival (months)	20.7 (2.4–102.9)	20.0 (8.1–64.2)

*P < 0.05 and ^#^ *P* < 0.1, when compared between TN and NAT.

All procedures were performed in accordance with the ethical standards of the institutional and/or national research committee and the Helsinki Declaration and its later amendments or comparable ethical standards. This study was approved by the Regional Ethics Committee of South‐Eastern Norway (REC South East, project number 2015/738). A written informed consent to use biomaterials and clinical information for research purpose was obtained from all participants included in this study.

### 2.3 Histopathological assessment

After collecting tissue samples for metabolomics, the surgical specimen was fixed in 10% neutral‐buffered formalin for 48 h and examined by an experienced pancreatic pathologist according to international guidelines [30]. Tumor regression grading for NAT‐treated PDACs was determined according to the College of American Pathologists (CAP) guidelines and categorized as CAP‐0 (complete response), CAP‐1 (near‐complete response), CAP‐2 (moderate response), CAP‐3 (poor response) [31]. The tissue block representing the tumor bed area from which the sample for metabolomics was taken was evaluated histologically to confirm the presence of tumor tissue.

### 2.4 Sample preparation

#### 2.4.1 Tissue

Each sample was homogenized to powder as described previously [18], following which samples were weighed, mixed with methanol (15 μL·mg^−1^ tissue) and water (6 μL·mg^−1^ tissue), vortexed for 20 s, and centrifuged at 4 °C, 20 000 ***g*** for 10 min. Methyl tert‐butyl ether (MTBE; 15 μL·mg^−1^ tissue) and water (7.5 μL·mg^−1^ tissue) were added and vortexed for 20 s, and the centrifugation step was repeated. Samples were first placed on ice for 5 min, and then in the refrigerator (4 °C) for 48 h to induce phase separation. Chloroform (15 μL·mg^−1^ tissue) was added to the mixture, which was subsequently vortexed for 20 s and centrifuged at 4 °C, 20 000 ***g*** for 10 min. Phase separation was completed by placing samples at room temperature for 30 min. The separated phases were transferred to two Eppendorf tubes: the top and bottom phase in one tube and the middle phase in the other tube. The middle phase was used for metabolomics analysis. Two hundred and fifty microliter of top and bottom phase mix was dried under a stream of nitrogen, following which 200 μL isopropyl alcohol (IPA) was added and mixed thoroughly.

#### 2.4.2 Serum

Serum samples were first thawed on ice. To 30 μL serum, 90 μL cold methanol was added and mixed, vortexed for 10 s, and centrifuged at 4 °C, 20 000 ***g*** for 10 min. The supernatant was collected for LC–MS analysis.

#### 2.4.3 PQC

To monitor the stability of the analysis and ensure the precision of the results, separate PQC samples were prepared for each individual sample group (tissue and serum). The PQC samples were prepared by transferring into a separate Eppendorf tube and mixing thoroughly a 30 μL aliquots of each sample in the respective group as well as a 30 μL aliquot of samples from both patients with pancreatic disease other than PDAC. All processed samples including the PQC samples were collected in separate HPLC vials with insert glass and cap for LC–MS analysis.

### 2.5 Metabolomic analysis

Samples were analyzed using Ultimate 3000 HPLC coupled to Q Exactive orbitrap mass spectrometer (Thermo Scientific, Waltham, MA, USA). In this study, full MS (of intact molecules for quantification) and MS/MS (fragmentation for metabolite identification) analysis were performed according to the procedure described by Skogvold et al. [32, 33], including identical parameters for liquid chromatography and electrospray ionization and MS settings. Samples were analyzed in both positive and negative ionization modes (in separate injections). Metabolites were separated using Pursuit XRs diphenyl column with gradient elution using mobile phase A: water + 0.01% formic acid and mobile phase B: methanol + 0.01% formic acid.

### 2.6 Data analysis

Data processing and statistical analysis were conducted using compound discoverer 3.3.1 (Thermo Scientific). The data processing incorporated retention time alignment and SERRF QC correction, among other operations. For more detailed information on data processing performed by compound discoverer, see workflow entitled ‘Untargeted Metabolomics with Statistics Detect Unknowns with ID Using Online Databases and mzLogic’ [34]. For the statistical analysis, compound discoverer calculated the *P*‐value per group ratio using a two‐tailed student's *t*‐test. Differential analysis in the form of volcano plots were used to compare both treatment groups (NAT and TN). Differentially accumulated metabolite features with *P* < 0.05 and log_2_ fold change < 0.5 or > 1 was considered statistically significant. Furthermore, *P*‐values were adjusted using the Benjamini–Hochberg correction method to account for false discovery rate (FDR). Unsupervised principal component analysis (PCA) was used to evaluate the analytical quality of the analysis and to visualize the overall metabolic profile of individual sample as well as variations among the samples.

Further data analysis including comparing both groups for altered metabolites, correlation analysis, and overall survival was carried out using the qlucore omics explorer 3.8.22 (Qlucore AB, Lund, Sweden). Correlation between metabolites and clinical parameters was assessed by using the non‐parametric Spearman's correlation test. Spearman's rank correlation coefficient (*r*) values of 0 to (±) 0.3, ± (0.3–0.7), and ± (0.7–1) were considered a weak, moderate, and strong positive/negative correlation, respectively. The correlation was considered statistically significant when *P* < 0.05, which was calculated using ANOVA. For identification of enriched pathways, all identified metabolites were first submitted to Metabolika pathways module available in the compound discoverer software. Next, the list of DAMs was submitted to metaboanalyst 6.0 [35], a web‐based tool to identify significantly enriched KEGG pathways with the level of statistical significance set at *P* < 0.05. To assess the performance of potential biomarkers a diagnostic model was constructed using MSI peak areas of five serum DAMs and CA 19‐9 values obtained from clinical assessment. Receiver operating characteristic (ROC) analysis was used to assess the accuracy of model predictions through determination of specificity and sensitivity, as well as by comparing the area under the curve (AUC) of single candidate biomarkers and their combinations using a binary logistic regression analysis. The ROC analysis was performed using Biomarker Analysis module of the MetaboAnalyst. ROC curves were generated using support vector machine (SVM) classifiers. Multivariate ROC exploratory analysis was used to identify the promising biomarkers with high sensitivity and specificity. For both univariate and multivariate statistical analyses, the level of statistical significance (*t*‐test) was set at *P* < 0.05.

## 3 Results

### 3.1 Characteristics of study participants

**Fig. 1:**
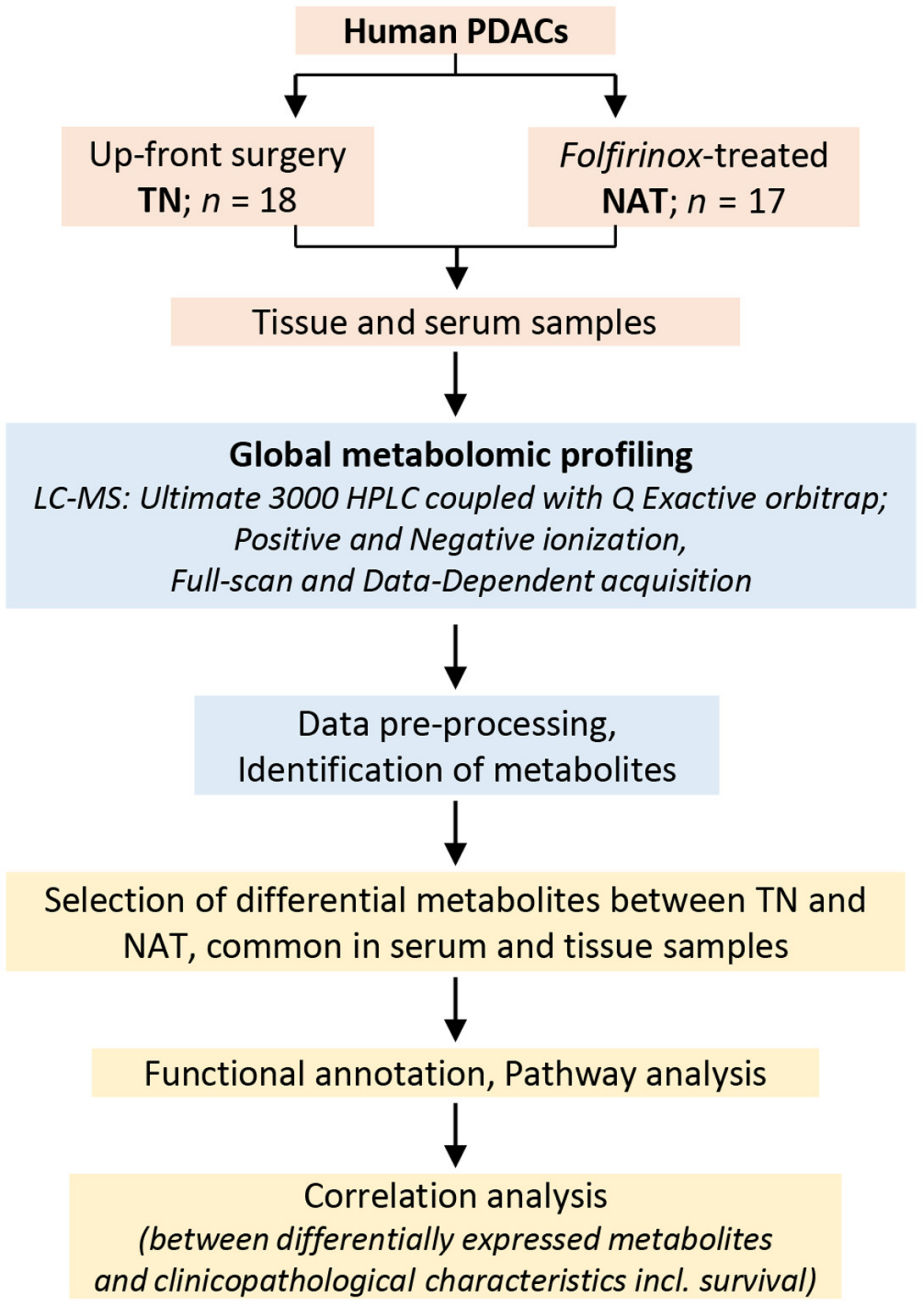
The study workflow. HPLC, high‐performance liquid chromatography; LC–MS, liquid chromatography mass spectrometry; NAT, neoadjuvantly treated; PDAC, pancreatic ductal adenocarcinoma; TN, treatment‐naïve.

The workflow of the study is provided in Fig. 1. Overall, 18 TN and 17 NAT (neoadjuvant FOLFIRINOX) PDAC tumors and matched serum samples were analyzed using LC–MS to explore metabolic differences between both treatment groups and to identify potential markers of treatment response in the NAT group. The 18 patients in the TN group (13 male, 5 female) had primary resectable tumors, whereas 11 of 17 in the NAT group (7 male, 10 female) had borderline resectable or locally advanced tumors. Patients in the NAT group were younger (median age 64 years) than those in the TN group (74.5 years; *P* < 0.05). The proportion of patients with comorbidity was lower in NAT (47%) compared to the TN group (83%). Detailed clinical information is provided in Table 1 and Table S1.

The median serum CA 19‐9 level in preoperative (after completion of chemotherapy) NAT samples (56 U·mL^−1^) was 5.2‐fold lower compared to both preoperative TN samples (290 U·mL^−1^) and pretreatment (before initiation of chemotherapy) NAT samples (289 U·mL^−1^). However, neither of these differences were statistically significant (*P* = 0.10 and *P* = 0.17, respectively). A trend towards lower serum C‐reactive protein (1.8‐fold, *P* = 0.07) was seen in NAT (2.4 mg·L^−1^) compared to TN samples (4.3 mg·L^−1^). Serum albumin levels were similar in both groups. Reflecting the effect of biliary stenting in the NAT group, bilirubin levels were significantly lower (6‐fold; *P* < 0.05) in NAT compared to TN samples (Table 1). Histopathological examination revealed a higher proportion of T3 tumors in TN (72%) compared to the NAT group (59%). Moreover, residual tumors in the NAT group showed moderate to poor histological response to neoadjuvant chemotherapy in all samples, corresponding to CAP‐2 (*n* = 8) and CAP‐3 (*n* = 9; Table 1). Representative histology images of TN and NAT tumors are shown in Fig. S1. Half of the patients in the TN group and almost all patients in the NAT group (16 of 17) received adjuvant chemotherapy. Median overall survival calculated from the date of diagnosis was 20.7 months for patients in the TN group (range: 2.4–102.9 months) and 20.0 months for patients in the NAT group (range: 8.1–64.2 months; Table S1, Fig. S2).

### 3.2 Untargeted metabolomics analysis

**Fig. 2:**
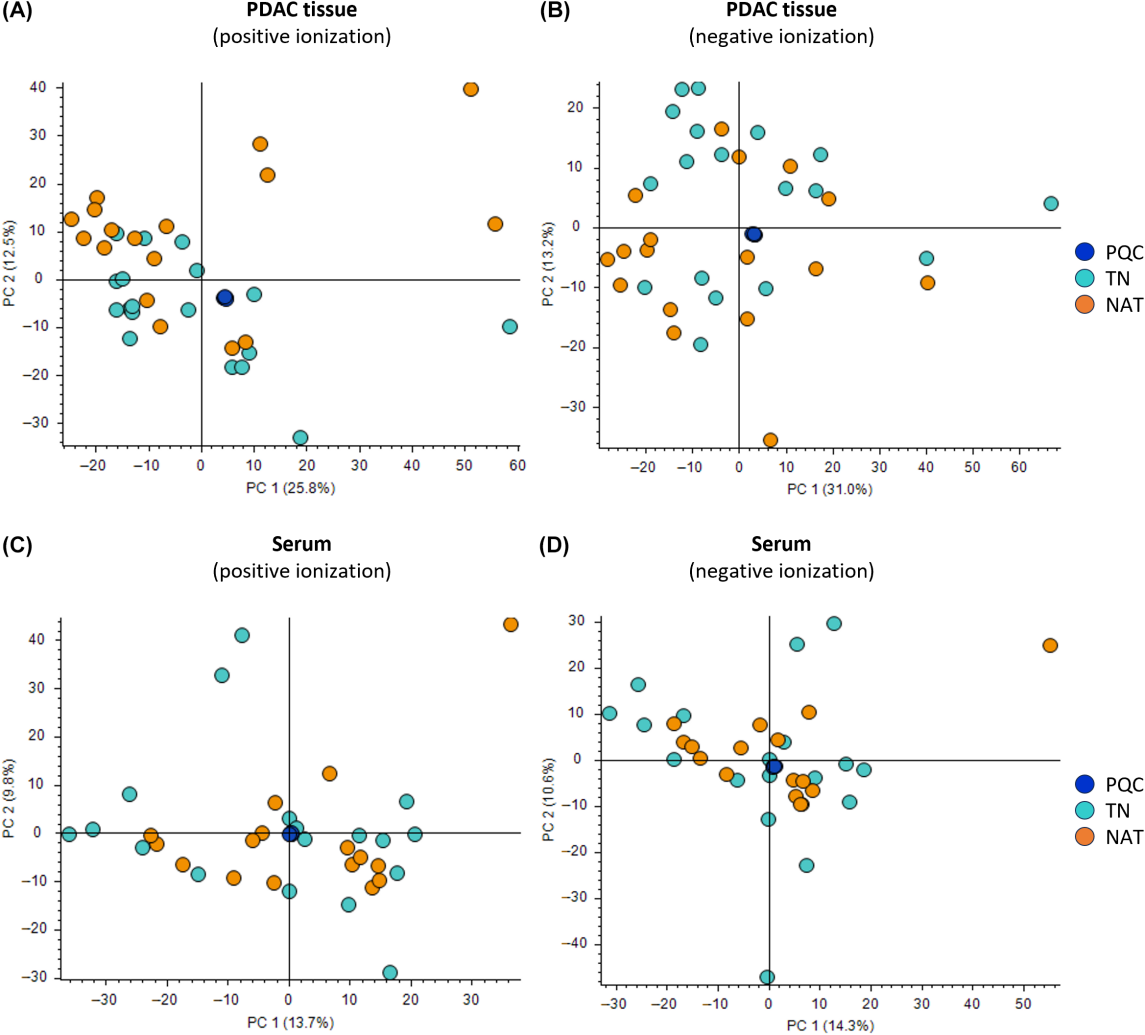
Principal component analysis (PCA). Untargeted metabolomics profiling of tumor tissue samples and paired serum samples obtained from 18 treatment‐naïve (TN) and 17 neoadjuvantly treated (NAT) pancreatic ductal adenocarcinoma (PDAC) patients. The PCA score plots for distribution for tissue samples (A, B) and serum samples (C, D), including pooled quality control (PQC) samples. *X*‐ and *Y*‐axis represent component 1 and 2, respectively.

Instrument stability and reliability of the data were confirmed using total ion chromatograms (TIC) of PQCs for both tissue and serum samples, which generally overlapped between positive and negative ionization modes (Fig. S3). In this study, unsupervised PCA was performed to visualize the overall distribution of metabolite features and their dispersion among all samples in both groups and in relation to PQCs. These PCA plots were generated based on all metabolite features detected and their respective peak areas in each individual sample. The PCA score plots showed a clear clustering of PQCs in both tissue samples (Fig. 2A,B) and serum samples (Fig. 2C,D), indicating the stability and the repeatability of the method. The PCA plots for both tissue and serum samples displayed heterogeneity between the samples within the same treatment group (TN or NAT) as well as between both groups. The variability among samples was larger in tissue samples compared to serum samples and this was likely driven by component PC 1 (Fig. 2). The PCA plots showed no obvious pattern that distinguished between the TN and NAT groups, neither in tissue nor in serum samples, and irrespective of the ionization mode.

**Fig. 3:**
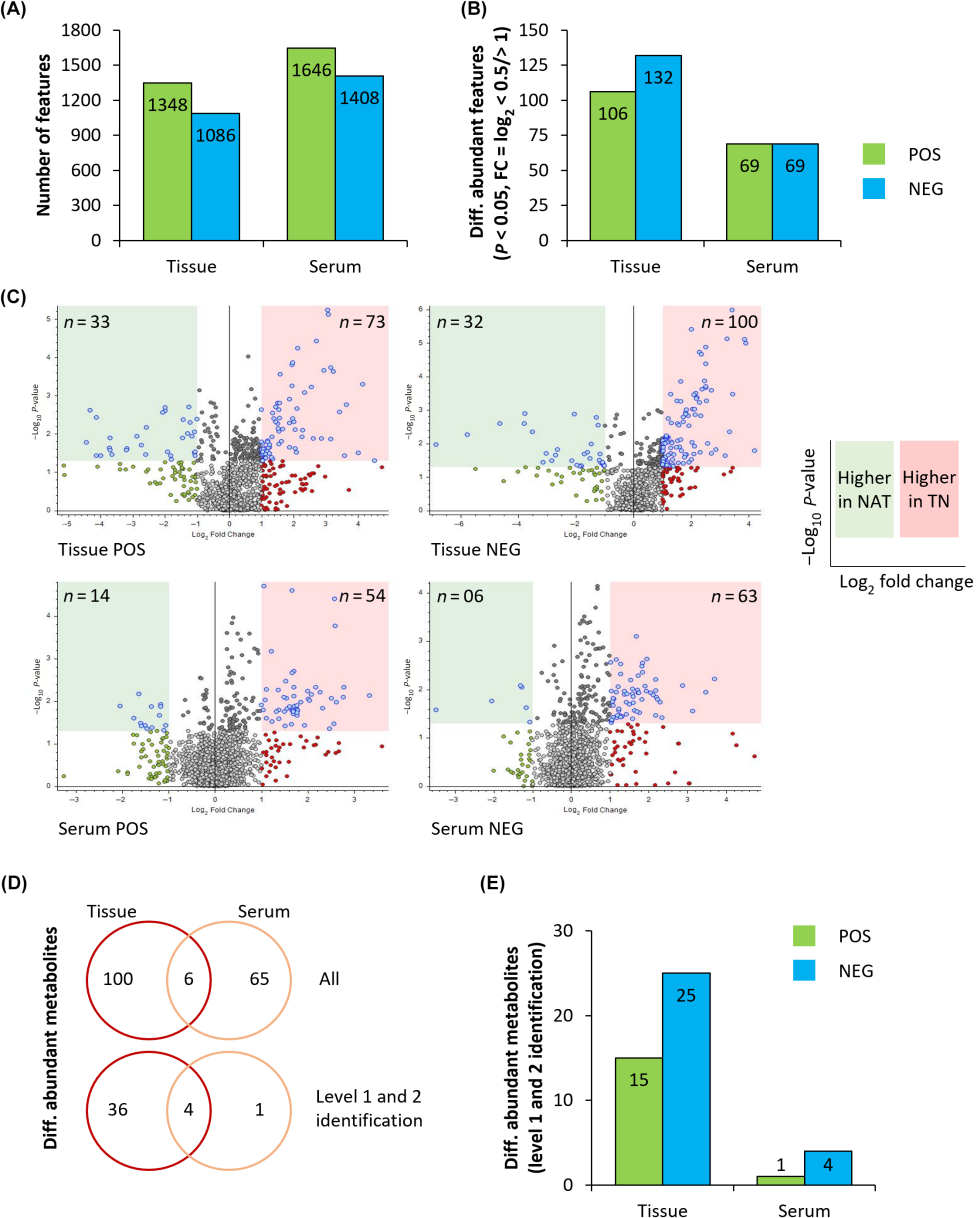
Overview of metabolomic profiles of PDAC tissue and serum samples. (A) Overview of all metabolic features found in tissue and serum samples in both positive (POS) and negative (NEG) ionization modes. (B) Number of differentially abundant metabolite (DAM) features between TN and NAT in both tissue and serum samples in both ionization modes. (C) Volcano plots showing distribution of DAM features according to their higher abundance in NAT (light green region) or TN (light red region) PDAC. (D) Venn diagram showing DAMs: all identified (upper panel), and those within level 1 and 2 identification (lower panel), and metabolites common to tissue and serum (upper and lower panel). (E) Overview of DAMs in TN versus NAT for both tissue and serum samples in both ionization modes. NAT, neoadjuvantly treated; PDAC, pancreatic ductal adenocarcinoma; TN, treatment‐naïve.

The total number of metabolite features detected by MS was 2434 for tissue samples and 3054 for serum samples (Fig. 3A). Comparing both ionization modes, a higher number of features was detected in the negative than in the positive mode, the difference being approximately 20% for tissue samples and 15% for serum samples. Of all the metabolite features detected (Fig. 3A), 238 (10%) and 138 (4.5%) features in tissue and serum samples, respectively, were differentially accumulated (*P* < 0.05 and log_2_‐fold change of < 0.5 or > 1) in the TN compared to the NAT groups (Fig. 3B). Of these, 106 and 132 features were detected in positive and negative ionization mode for tissue samples, respectively, and 69 features each in either mode for serum samples. The distribution of differentially abundant metabolite (DAM) features in the TN versus NAT groups is presented as volcano plots in Fig. 3C. Interestingly, the proportion of metabolites with relatively lower abundance in NAT compared to TN samples (i.e., higher amounts in the TN group) is larger in both tissue samples and serum samples, as well as in both ionization modes (Fig. 3C).

**Fig. 4:**
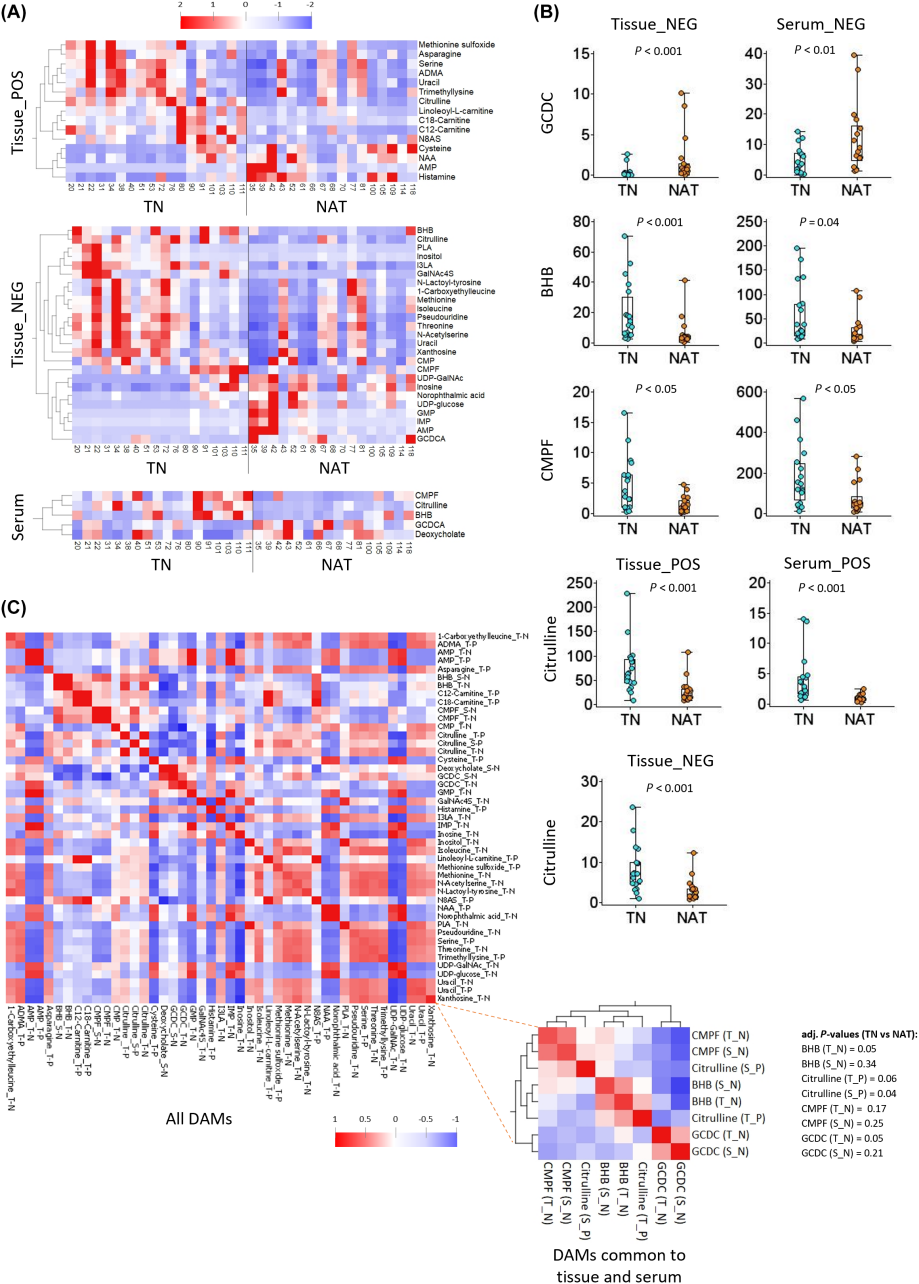
Differentially abundant metabolites (DAMs) in TN versus NAT groups. (A) Heatmaps showing pattern of DAMs detected in positive (POS) and negative (NEG) ionization mode for tissue samples and combined for serum samples. (B) Box and scatter plots for four DAMs identified in both tissue and serum data. Error bars represent standard deviation, and the statistical significance between groups were determined using student's *t*‐test. (C) Heatmap showing correlation between all DAMs and between the four DAMs common to tissue (T_N, T_P) and serum (S_N, S_P). N and P represent negative and positive ionization mode, respectively. ADMA, *N*,*N*‐dimethylarginine; AMP, adenosine monophosphate; BHB, 3‐hydroxybutyric acid; CMP, cytidine monophosphate; CMPF, 3‐carboxy‐4‐methyl‐5‐propyl‐2‐furanpropanoic acid; GalNAc4S, *N*‐acetyl‐d‐galactosamine 4‐sulfate; GCDC, glycochenodeoxycholate; GMP, guanosine monophosphate; I3LA, indole‐3‐lactic acid; IMP, inosine monophosphate; N8AS, N8‐acetylspermidine; NAA, *N*‐acetylaspartate; NAT, neoadjuvantly treated; PLA, phenyllactic acid; TN, treatment‐naïve; UDP‐GalNAc, uridine‐diphosphate‐*N*‐acetylgalactosamine.

Next, all DAM features were processed for identification and annotation. This identified a total 106 and 71 metabolites in tissue and serum samples, respectively, that were differentially abundant in TN versus NAT. Subsequently, these metabolites were further filtered by comparing their mass spectrum profiles with the in‐house metabolite library (level 1 identification) and with public databases, including the Human Metabolome Database (HMDB; level 2 identification; Fig. 3D). This reduced the number of unique metabolites with differential abundance in the TN versus NAT group to 40 for tissue samples and 5 for serum samples (Fig. 3D,E). A complete list of these 45 metabolites is provided in Table 2 and associated raw data is provided in Table S2. These belong to diverse metabolite categories, including amino acids, bile salts, purine and pyrimidine nucleosides, amines, fatty acid esters, and carboxylic sugars. Of the 40 DAMs identified in TN versus NAT tissue samples, 15 and 25 were detected by positive and negative ionization modes, respectively (Fig. 3E). Three tissue metabolites were detected in both ionization modes: adenosine monophosphate (AMP), citrulline, and uracil. Heatmaps showing the abundance of DAMs in tissue and serum samples are provided in Fig. 4A.

**Table 2:** List of differentially abundant metabolites in the TN versus NAT groups. Metabolites in positive and negative ionization are indicated with normal and italics font, respectively. Adjusted *P*‐values account for the Benjamini–Hochberg correction procedure for false discovery. CMPF, 3‐carboxy‐4‐methyl‐5‐propyl‐2‐furanpropanoic acid; FC, fold change; GalNAc4S, *N*‐acetyl‐d‐galactosamine 4‐sulfate; UDP‐GalNAc, uridine‐diphosphate‐*N*‐acetylgalactosamine.

Metabolite name	Metabolite category	*m/z*	Level	TN vs NAT		
				FC	*P*‐value	Adj. *P*‐value
Tissue						
Histamine	Biogenic amine	112.0868	1	0.3	0.00**	0.17
N8‐Acetylspermidine	Polyamine	188.1756	2	2.9	0.01*	0.26
*N‐Acetylserine*	Amino acid	146.0460	1	2.3	0.03*	0.26
*N‐Lactoyl‐tyrosine*	Amino acid	252.0878	2	2.5	0.02*	0.21
*N*‐Acetylaspartate	Amino acid	176.0552	1	0.5	0.04*	0.36
*N*,*N*‐Dimethylarginine	Amino acid	203.1500	1	2.2	0.04*	0.38
*1‐Carboxyethylleucine*	Amino acid	202.1087	2	2.0	0.01*	0.17
*Leucine isomers*	Amino acid (essential)	130.0874	1	2.1	0.04*	0.28
*Methionine*	Amino acid (essential)	189.1596	2	2.2	0.03*	0.25
Methionine sulfoxide	Amino acid (essential)	166.0531	2	3.0	0.00**	0.06^#^
Trimethyllysine	Amino acid (essential)	118.0511	1	4.2	0.03*	0.35
*Threonine*	Amino acid (essential)	148.0440	1	2.0	0.02*	0.20
Asparagine	Amino acid (non‐essential)	174.0885	1	2.1	0.03*	0.36
Citrulline	Amino acid (non‐essential)	176.1028	1	3.9	0.00**	0.06^#^
*Citrulline*	Amino acid (non‐essential)	133.0607	1	4.4	0.00**	0.02*
Cysteine	Amino acid (non‐essential)	106.0498	2	0.1	0.02*	0.33
Serine	Amino acid (non‐essential)	122.0269	1	2.2	0.02*	0.33
*Glycochenodeoxycholate*	Bile salt	448.3071	1	0.2	0.00**	0.05*
*Inositol*	Carbocyclic sugar	179.0351	1	2.8	0.00**	0.03*
C18‐Carnitine	Fatty acid ester	428.3726	1	2.4	0.02**	0.33
C12‐Carnitine	Fatty acid ester	344.2786	2	4.3	0.00**	0.04*
Linoleoyl (C18:2) carnitine	Fatty acid ester	424.3414	2	3.0	0.00**	0.20
*CMPF*	Furan fatty acid	239.0927	1	3.0	0.01*	0.17
*3‐Hydroxybutyric acid*	Ketone body	103.0401	1	4.1	0.00**	0.05*
*Indole‐3‐lactic acid*	Monocarboxylic acid	204.0668	2	4.0	0.00**	0.00*
*Phenyllactic acid*	Monocarboxylic acid	165.0559	2	14.4	0.00**	0.00*
*Pseudouridine*	Nucleotide sugar	243.0622	2	3.1	0.00**	0.03*
*UDP‐GalNAc*	Nucleotide sugar	606.0747	2	0.0	0.01*	0.15
*Uridine diphosphate glucose*	Nucleotide sugar	565.0486	2	0.0	0.00**	0.07^#^
Adenosine monophosphate	Purine ribonucleoside	283.0684	1	0.1	0.00**	0.20
*Adenosine monophosphate*	Purine ribonucleoside	267.0734	1	0.1	0.00**	0.11
*Guanosine monophosphate*	Purine ribonucleoside	347.0402	1	0.1	0.00**	0.07^#^
*Inosine*	Purine ribonucleoside	362.0510	1	0.3	0.05*	0.28
*Inosine monophosphate*	Purine ribonucleoside	346.0561	1	0.1	0.03*	0.26
*Xanthosine*	Purine ribonucleoside	348.0695	1	3.1	0.01*	0.14
Uracil	Pyrimidine nucleobase	111.0201	1	2.8	0.00**	0.17
*Uracil*	Pyrimidine nucleobase	113.0344	1	3.3	0.00**	0.07^#^
*Cytidine monophosphate*	Pyrimidine ribonucleoside	322.0448	1	2.2	0.02*	0.23
*GalNAc4S*	Sugar	300.0396	2	4.6	0.00**	0.03*
*Norophthalmic acid*	Tripeptide	274.1044	2	0.2	0.02*	0.21
Serum						
Citrulline	Amino acid (non‐essential)	176.0665	1	3.2	0.00**	0.04*
*Deoxycholate*	Bile salt	391.2855	1	0.4	0.02*	0.29
*Glycochenodeoxycholate*	Bile salt	448.3069	1	0.4	0.01*	0.21
*CMPF*	Furan fatty acid	239.0925	1	2.7	0.02*	0.25
*3‐Hydroxybutyric acid*	Ketone body	103.0400	1	2.3	0.04*	0.34

^#^ *P* < 0.1, *P < 0.05, **P < 0.01 between treatment‐naïve (TN) and neoadjuvantly treated (NAT) samples.

Next, unique DAMs detected both in tissue and serum samples were identified (Fig. 3D). These included glycochenodeoxycholate (GCDC), 3‐hydroxybutyric acid (also known as β‐hydroxybutyric acid, BHB), 3‐carboxy‐4‐methyl‐5‐propyl‐2‐furanpropanoic acid (CMPF), and citrulline. BHB, citrulline, and GCDC had an adjusted *P*‐value below 0.05 following Benjamini–Hochberg correction. The profiles of their relative abundance in the TN and NAT groups are presented as boxplots in Fig. 4B. GCDC, a bile salt, was significantly higher in both tissue and serum samples in the NAT compared to the TN group. BHB (a ketone body), CMPF (a furan fatty acid), and citrulline (a precursor for arginine biosynthesis) were significantly lower in both tissue and serum samples in the NAT compared to the TN group (Fig. 4B). GCDC, BHB, and CMPF were detected in negative ionization mode in both tissue and serum samples. Citrulline was detected in both positive and negative ionization mode in tissue samples (Fig. 4B).

Next, as amino acids are important substrates, byproducts, and key players in tumor metabolic processes, and their metabolism, mainly of essential branched‐chain amino acids has a central role in many cancers and particularly in PDAC [36], tissue and serum metabolome datasets were searched for the commonly known 9 essential and 11 non‐essential amino acids (Table 3). Six of these were found with significantly different abundance in TN versus NAT tissue samples. Abundance of leucine isomers (isoleucine, alloisoleucin and leucine), methionine, threonine, asparagine, proline, and serine were significantly lower in NAT compared to TN. Only four amino acids showed different abundance when comparing TN and NAT serum samples: histidine, lysine, threonine, and cystine. Except cystine, the other three were significantly higher in NAT compared to TN. Moreover, a trend towards different abundance of histidine, phenylalanine, tryptophan, cystine, and tyrosine was seen in tissue samples, but the differences did not reach statistical significance (*P* < 0.1; Table 3).

**Table 3:** Relative abundance of the most common amino acids in the TN versus NAT PDACs. As leucine, alloisoleucin, and isoleucine could not be separated chromatographically in this analysis, they were considered as leucine isomers. NAT, neoadjuvantly treated; ND, not detected; NEG, negative ion mode; POS, positive ion mode; TN, treatment‐naïve.

TN/NAT ratio	Tissue POS	Tissue NEG	Serum POS	Serum NEG	Identification level
Essential amino acids					
Histidine	1.5^#^	1.2	0.9*	0.9	1
Leucine isomers	1.6*	2.1*	1.0	1.0	1
Lysine	1.1	1.0	0.9	0.8*	1
Methionine	1.9*	2.2*	0.8	ND	1
Phenylalanine	1.6^#^	1.8^#^	1.1	1.1	2
Threonine	ND	2.0*	ND	0.9*	1
Tryptophan	1.6^#^	1.7^#^	1.0	1.0	1
Valine	1.3	ND	0.9	ND	1
Non‐essential amino acids					
Alanine	1.0	ND	0.9	ND	1
Arginine	1.2	1.0	0.9	0.9	1
Asparagine	2.1*	ND	1.0	1.0	1
Aspartate	0.9	0.7	1.1	1.1	1
Cystine	1.7^#^	1.8^#^	1.2*	1.2*	1
Glutamate	0.9	1.0	1.2	1.2	1
Glutamine	0.9	1.1	0.9	0.8	1
Glycine	0.9	ND	1.0	ND	1
Proline	1.8*	ND	1.0	ND	1
Serine	2.2*	2.0*	1.1	1.1	1
Tyrosine	1.7^#^	1.7^#^	1.0	1.0	1

*P < 0.05 indicates animo acids with statistically significant differences between both groups while ^#^ *P* < 0.1 indicates a trend towards altered metabolite levels between both groups.

### 3.3 Correlation between metabolites

The pattern of differential abundance in the TN versus NAT groups was similar for the four metabolites common to tissue and serum samples (Fig. 4B, Table 2). Next, spearman's correlation analysis was used to evaluate the correlation between a given pair of DAMs including between four DAMs common to tissue and serum. Their heatmaps are presented in Fig. 4C. As shown in Fig. 4C, a total of 37 out of 45 DAMs showed significantly strong correlation (*r* > 0.7, *P* < 0.05) with at least one other DAM. Eight DAMs showed no significant correlation with other DAMs: serum citrulline, deoxycholate and GCDC, and tissue CMP, cysteine, GalNAc4S, GCDC, and histamine. Tissue uracil was found to strongly correlate with 18 other DAMs, followed by tissue pseudouridine and serine that both showed strong correlation with 14 other DAMs. The correlation coefficients (*r*) for the various comparisons for four common DAMs are provided in Table S3. GCDC (*r* = 0.36, *P* < 0.05), BHB (*r* = 0.72, *P* < 0.01), and CMPF (*r* = 0.83, *P* < 0.01) correlated positively between tissue and serum. A weak negative correlation (*r* = 0 to −0.3) was seen between tissue GCDC and the other three metabolites in both tissue and serum. Serum GCDC correlated moderately negatively (*P* < 0.05) with serum BHB (*r* = −0.35) and CMPF (*r* = −0.36) and with tissue CMPF (*r* = −0.42). Tissue BHB correlated positively with tissue citrulline (*r* = 0.52 and 0.54, *P* < 0.01) and serum BHB correlated positively (*P* < 0.05) with citrulline and CMPF in both serum and tissue.

### 3.4 Correlation between levels of metabolites and clinical parameters

**Fig. 5:**
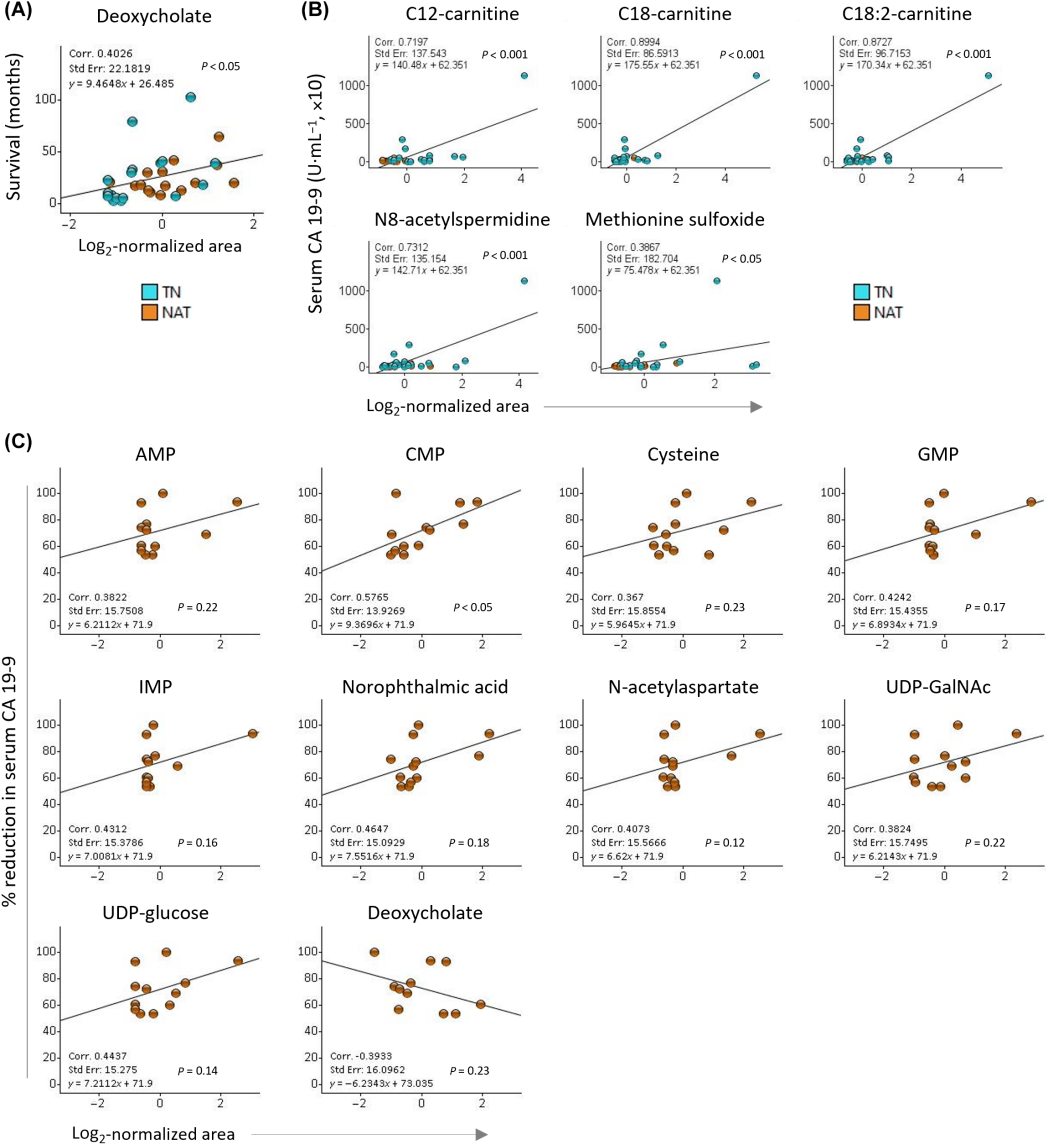
Correlation analysis. Scatter plots showing correlation between the amounts of metabolites on *x*‐axis and (A) survival, (B) serum carbohydrate 19‐9 antigen (CA 19‐9) levels in both TN and NAT samples, and (C) percentage reduction in CA 19‐9 levels following neoadjuvant treatment (NAT samples) on *y*‐axis. The correlation significances were calculated using ANOVA. AMP, adenosine monophosphate; CMP, cytidine monophosphate; GMP, guanosine monophosphate; IMP, inosine monophosphate; NAT, neoadjuvantly treated; TN, treatment‐naïve; UDP‐GalNAc, uridine‐diphosphate‐*N*‐acetylgalactosamine; UDP‐glucose, uridine‐diphosphate‐glucose.

Of the 45 DAMs in the TN versus NAT group, only serum deoxycholate showed moderate positive correlation (*r* = 0.4, *P* < 0.05) with overall survival (Fig. 5A). No correlation was found between the tissue DAMs and survival. Interestingly, five tissue metabolites correlated positively with serum CA 19‐9: C12‐carnitine, C18‐carnitine, linoleoyl (C18:2)‐carnitine, and N8‐acetylspermidine (N8AS) showed strong correlation (*r* > 0.7, *P* < 0.001), while methionine sulfoxide showed moderate correlation (*r* = 0.39, *P* < 0.05; Fig. 5B). No correlation was found between the level of serum metabolites and CA 19‐9 levels. Serum C‐reactive protein (CRP) and tumor size correlated moderately positively with both serum and tissue BHB levels (Fig. S4). Both clinical parameters also showed moderate correlation with other metabolites. CRP correlated positively with tissue citrulline, cytidine monophosphate, CMPF, inosine, N8AS, and *N*‐acetylaspartate and negatively with serum deoxycholate and GCDC. Tumor size correlated positively with tissue CMPF, N8AS, and uridine‐diphosphate‐*N*‐acetylgalactosamine (UDP‐GalNAc; Fig. S4). Median age, which was lower for patients in the NAT group than for those in the TN group correlated only with CMPF in both tissue and serum (moderate correlation, *r* = 0.3–0.7, *P* < 0.05; Fig. S4).

### 3.5 Identification of potential markers of treatment response

Next, to identify potential markers of treatment response, the correlation between the 45 DAMs in TN versus NAT group and the change in CA 19‐9 level between pretreatment and preoperative blood samples for patients in the NAT group (*n* = 17) was analyzed. Pretreatment serum CA 19‐9 data was accessible for 15 patients, and 12 of these showed > 50% reduction in the level of CA 19‐9 following NAT. The remaining three patients showed no change or increased level of CA 19‐9 (Table S1). Correlation analysis of the DAMs in the samples with reduced CA 19‐9 following NAT identified 10 metabolites with a moderate correlation. It revealed one serum metabolite – deoxycholate – with negative correlation and 9 tissue metabolites with positive correlation, including AMP, cytidine monophosphate (CMP), guanosine monophosphate (GMP), inosine monophosphate (IMP), cysteine, norophthalmic acid, *N*‐acetylaspartate, UDP‐GalNAc, and UDP‐glucose (Fig. 5C). Furthermore, the correlation between DAMs and change in CA 19‐9 following NAT, was analyzed, including for patients with increased CA 19‐9. Eight tissue and two serum metabolites were identified: moderate positive correlation was observed for tissue AMP, CMP, GMP, and C12‐carnitine as well as serum CMPF, while moderate negative correlation was found for tissue asparagine, citrulline, inositol, and phenyllactic acid as well as serum BHB (Fig. S5).

**Fig. 6:**
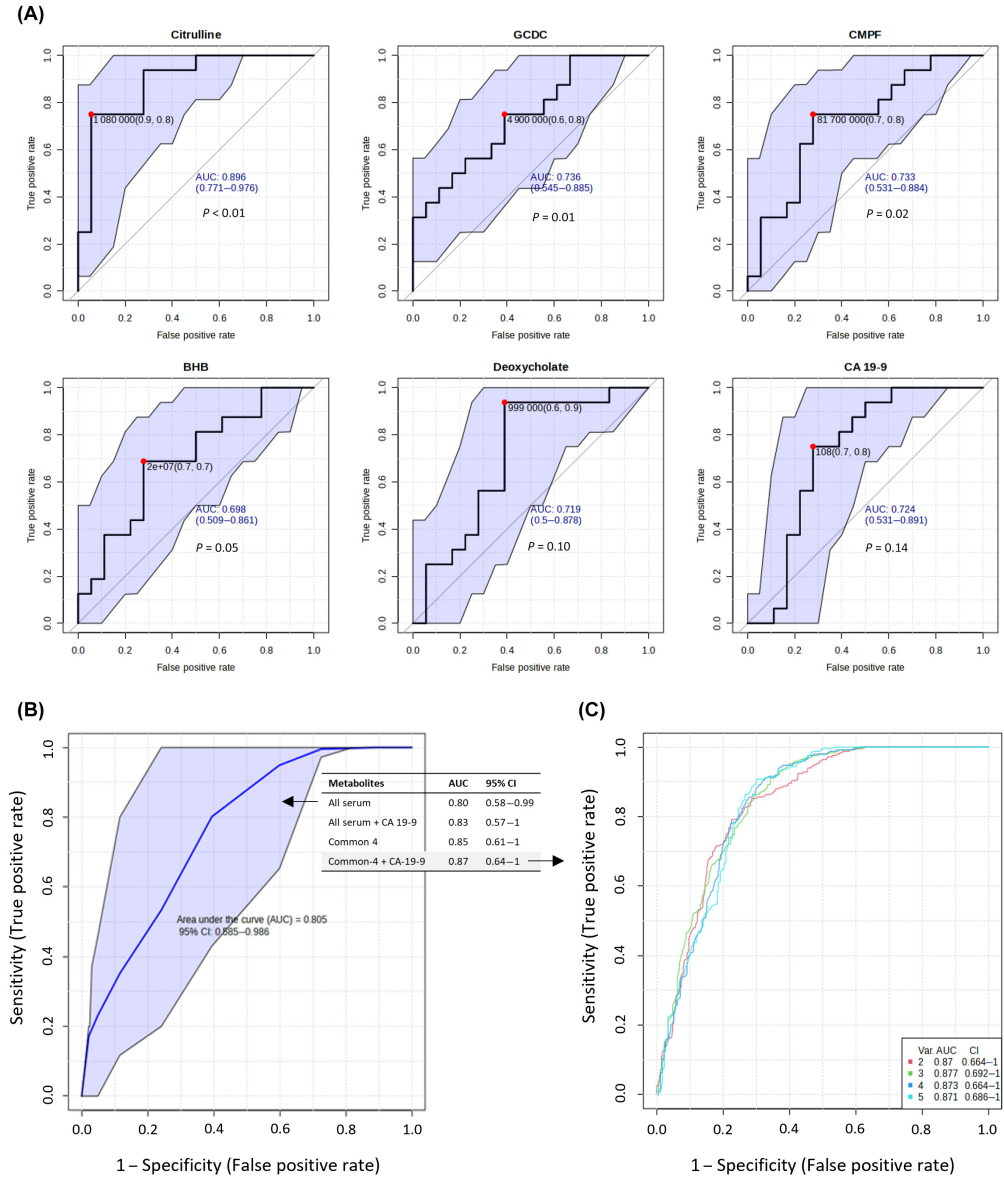
Assessment of diagnostic performance. (A) Receiver operating characteristic (ROC) curves generated in Biomarker Module of MetaboAnalyst for five serum DAMs and carbohydrate 19‐9 antigen (CA 19‐9). The computed 95% confidence interval (CI) for individual marker metabolites is highlighted in the light purple background. The area under the ROC curve (AUC) is shown in blue to highlight the diagnostic potential of the respective metabolite. A Red dot closest to the top‐left corner represents the optimal cutoff point. Statistical significances were determined using standard *t*‐test. (B) ROC curve computed for all five serum DAMs with the 95% CI. (C) Multivariate ROC analysis showing the feature numbers (var), AUCs, and CIs of the five models for BHB, CMPF, citrulline, GCDC, and CA 19‐9. Numbers 2–5 represent different models with the respective number of features. BHB, 3‐hydroxybutyric acid; CMPF, 3‐carboxy‐4‐methyl‐5‐propyl‐2‐furanpropanoic acid; DAMs, differentially abundant metabolites; GCDC, glycochenodeoxycholate.

The diagnostic performance of all five serum DAMs as potential biomarkers of treatment response was evaluated based on the area under ROC curves in NAT versus TN samples. ROC curves for the individual DAM and for CA 19‐9 are provided in Fig. 6A. Among these five DAMs, citrulline showed the highest discriminatory potential with AUC = 0.90 and 95% CI = 0.77–0.98. All five DAMs showed AUC ≥ 0.7, and except for deoxycholate the other four DAMs, which were also common to tissues, showed *P* < 0.05. A better prediction was observed individually for citrulline, GCDC, and CMPF compared to CA 19‐9, which had AUC = 0.72 and 95% CI = 0.53–0.89, but the difference was not statistically significant. Next, we attempted to improve the prediction using a multivariate ROC curve exploratory analysis. First, a ROC curve was generated for all five serum DAMs (Fig. 6B), which resulted in AUC = 0.80 and 95% CI = 0.58–0.99. Next, a multivariate ROC curve analysis was performed involving a combination of more than one discriminatory metabolite selected via logistic regression analysis. AUC and CI for different combinations are provided in tabular format in Fig. 6B,C. Among several models, combination of CA 19‐9 with the four DAMs common to serum and tissue produced the highest AUC 0.87 and 95% CI = 0.64–1. Figure 6C shows an overview of all ROC curves created from five different biomarker models using different number of features, excluding deoxycholate.

### 3.6 Pathway analysis

**Fig. 7:**
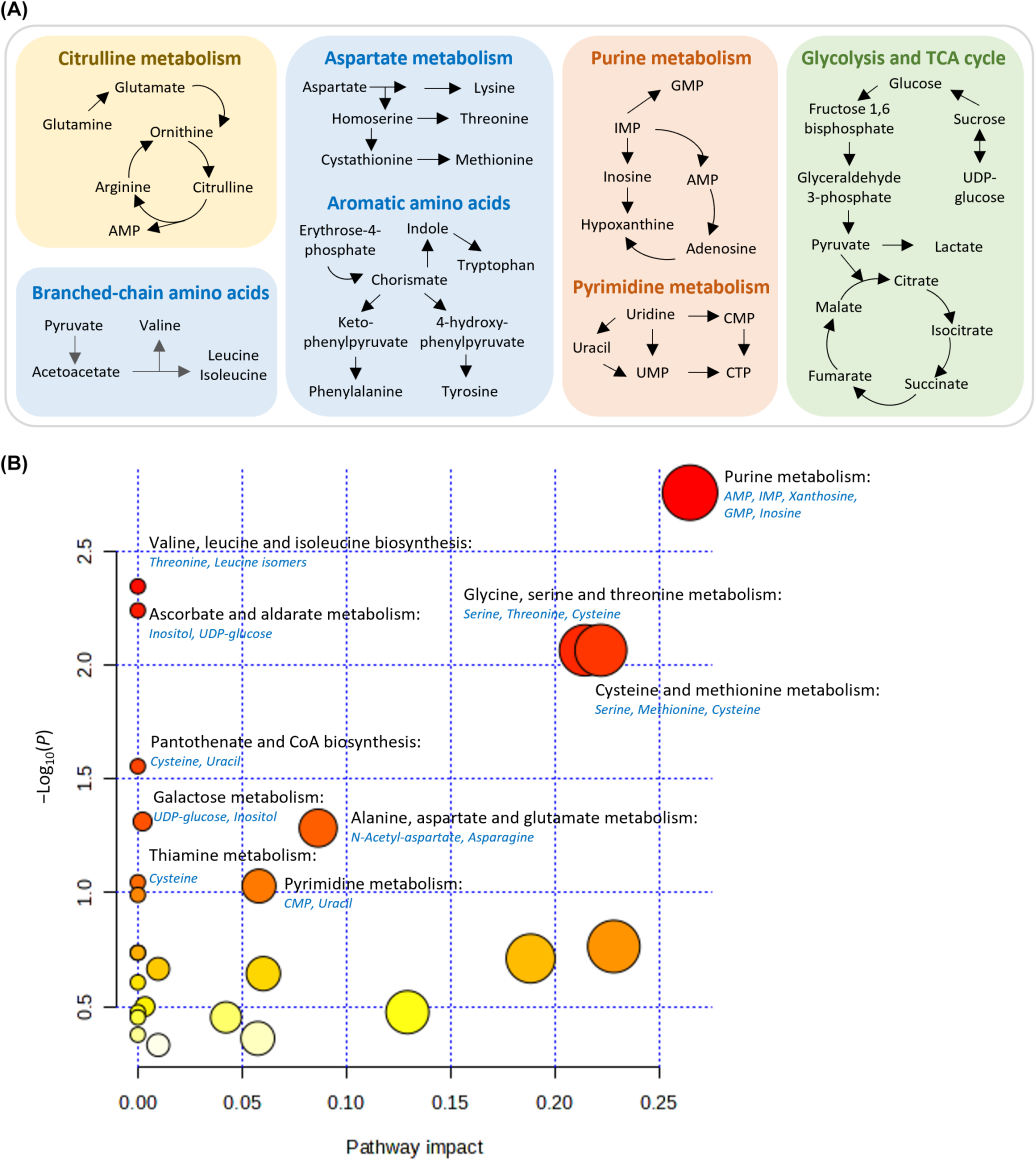
Pathway analysis. Most enriched pathways (A) for all metabolites identified in the pancreatic cancer metabolome and (B) for differentially abundant metabolites (DAMs) in neoadjuvantly treated versus treatment‐naive samples. In (B), pathways are arranged based on both the significance of enrichment, i.e., *P*‐values (*y*‐axis), and pathway impact (topology analysis; *x*‐axis). The node color is determined by the *P*‐value (red = highest statistical significance), while the node size represents pathway impact factor (larger size = stronger impact). DAMs are indicated in the parentheses in blue colored text. AMP, adenosine monophosphate; CMP, cytidine monophosphate; CTP, cytidine triphosphate; GMP, guanine monophosphate; IMP, inosine monophosphate; UDP, uridine diphosphate; UMP, uridine monophosphate.

To visualize the most abundant metabolic pathways associated with the metabolome identified the Metabolika module was used. The top 10 pathways with the maximum number of hits and relevance to the study were selected (Table S4). These included the salvage of purines and pyrimidines, citrulline metabolism, biosynthesis of branched‐chain and aromatic amino acids, aspartate metabolism, biosynthesis of arginine and polyamines, glycolysis and TCA cycle, anaerobic sucrose degradation, and lysine degradation (Fig. 7A). Next, a list of DAMs in TN versus NAT was deployed to MetaboAnalyst to determine the enrichment of KEGG pathways. Seven significantly enriched pathways (*P* < 0.05) with diverse metabolite groups were identified. These included the purine metabolism, biosynthesis of valine, leucine, and isoleucine, ascorbate and aldarate metabolism, metabolism of amino acids including glycine, serine, threonine, cysteine and methionine, pantothenate and CoA biosynthesis, and galactose metabolism (Fig. 7B). In addition, three pathways showed a trend towards significant enrichment (*P* < 0.1): alanine, aspartate and glutamate metabolism, thiamine metabolism, and pyrimidine metabolism (Fig. 7B).

## 4 Discussion

During the past decades, the introduction of combination chemotherapy and neoadjuvant treatment (NAT) has increased treatment options for PDAC. Indeed, NAT is currently considered standard treatment for borderline and locally advanced disease, while for primary resectable PDAC its benefits are still debated [3, 8, 9, 16]. However, these therapeutical developments have not translated into significantly improved patient survival [9], the root causes of which are deemed to be molecular intratumor heterogeneity and rapid development of chemoresistance [37]. In addition, the lack of methods that enable early and accurate evaluation of treatment response limits the possibility to optimize chemotherapy. Thus, there is a need for biomarkers to evaluate treatment effect both accurately and early, and in this respect advanced high‐throughput omics techniques seem to have great potential [18, 19, 38, 39]. Our recent study on PDAC tissue proteome profiles revealed the impact of NAT on tumor and systemic metabolism in PDAC patients, as evidenced by the markedly lower expression of metabolism‐related proteins in NAT compared to TN patients [18]. To further investigate the impact of chemotherapy on tumor and systemic metabolism in PDAC patients, global metabolomic profiling of tumor and matched serum samples from neoadjuvant FOLFIRINOX‐treated and TN PDACs was undertaken. Global metabolomics is considered a hypothesis‐generating approach, which identifies all possible metabolites in a biological sample, including unknown metabolites, not just a pre‐defined list of metabolites. Only metabolites that passed level 1 (validated) and level 2 (putatively annotated) identification were selected for comparative analysis between both groups [40]. Metabolites belonging to level 1 were fully validated in‐house by confirming their structure using a reference standard while level 2 metabolites were identified through their structure match using fragmentation data from metabolite libraries and public databases such as MassBank of North America (MoNA).

In the present study, metabolomic profiling revealed multiple metabolic features that differed between the NAT and TN groups, corresponding to 106 tissue and 71 serum metabolites. Further data processing and annotation identified a total of 40 unique DAMs in TN versus NAT PDACs. The majority of these are related to amino acid metabolism as well as nucleotide biosynthesis and metabolism. The DAMs identified in this study do not directly correspond to the differentially expressed proteins following NAT as described in our recent study [18] or in the study by Stillger et al. [19], which found lower expression of fatty acid oxidation proteins and increased ribosomal factors following NAT. These apparent inconsistencies suggest that combined proteomic and metabolic analyses may be needed to understand the effect of therapy.

Metabolic reprogramming is a hallmark of PDAC, whereby cancer cells may acquire nutrients from metabolic pathways other than the glucose metabolism, in particular the amino acid and lipid metabolism [41, 42]. Six amino acids differed significantly in tissue samples, and notably, all these were lower in NAT compared to TN. While for serum samples, three of the four significantly altered animo acids were higher in NAT compared to TN. Branched‐chain amino acids—leucine, isoleucine, and valine—represent a subclass of amino acids, whose uptake and transamination is induced by KRAS, the most common mutation in PDAC [43, 44]. Interestingly, while valine was not affected by NAT, abundance of leucine isomers (isoleucine, alloisoleucin and leucine) was nearly 2‐fold lower in the NAT group. This suggests that NAT induces distinct branched‐chain amino acid metabolism in addition to that induced by KRAS. Amino acids are pivotal to the synthesis of cellular building blocks and to nucleotide metabolism, both of which support tumor growth [41, 42]. Most purine metabolites were more abundant, while pyrimidine metabolites were less abundant in NAT compared to TN tumors. To gather nutrients from lipid metabolic pathways, carnitines—amino acid‐derived compounds—are essential cofactors [45]. Three carnitines—C12, C18, and C18:2—were markedly lower in NAT compared to TN tumors. Overall, NAT tumors showed lower levels of metabolites related to amino acid metabolic pathways, an altered nucleotide metabolism, and reduced carnitines. Altogether these findings may indicate chemotherapy‐induced metabolic suppression in the tumor, which is in line with the proteomics findings from our recent study [18].

Five unique DAMs were identified in TN versus NAT serum samples, and interestingly, four of these—GCDC, BHB, citrulline, and CMPF—were also identified in tumor tissue samples. GCDC was higher, while BHB, citrulline and CMPF were lower in NAT compared to TN in both serum and tumor samples. The serum bile acids—GCDC and deoxycholate—were significantly higher in the NAT compared to the TN group. While the role of bile acids is still unclear and some evidence indicates that they may suppress cell proliferation, their biological significance with respect to NAT is currently unknown [46, 47, 48]. BHB is one of the main ketone bodies, which acts as a cellular endogenous or systemic fuel to promote the growth and progression of PDAC [49, 50]. Citrulline is a precursor for arginine biosynthesis, which is upregulated in many cancers including PDAC [51, 52]. The role of CMPF—a furan fatty acid—in PDAC is not clearly known. However, it has been reported to be involved in β‐cell dysfunction, insulin resistance, and apoptosis in kidney cells [53, 54, 55].

Overall survival of patients in the NAT group did not significantly differ from that of patients in the TN group. No strong correlation was found between tumor or serum DAMs and survival, only deoxycholate correlated moderately positively with survival. However, serum CA 19‐9 correlated strongly positively with four tissue metabolites, including carnitines (C12, C18, and C18:2), and polyamine N8AS, and all these were lower in NAT compared to TN. Carnitines facilitates the transport of acyl groups for mitochondrial β‐oxidation and play a crucial role in cancer cells' metabolic plasticity [56]. Carnitine deficiency has been reported in multiple cancers including advanced PDAC, which contributes to cachexia and thereby affecting the patients' quality of life [45, 57]. N8AS was recently suggested to be a potential metabolite biomarker for PDAC that is likely derived from cancer‐associated fibroblasts [58].

As one of the aims of this study was to identify potential markers of treatment response, we also investigated the correlation between CA 19‐9 change and metabolite abundance in the NAT group alone. CA 19‐9 was not reduced in all patients following NAT, and it remained unchanged or increased compared to pretreatment levels in three patients. For patients with markedly reduced CA 19‐9 following NAT, the change in CA 19‐9 correlated moderately positively with nine tissue metabolites and negatively with serum deoxycholate. Among tissue metabolites, six contribute to nucleotide metabolism and three to amino acid metabolism. Furthermore, only five serum metabolites were differentially abundant between NAT versus TN. We investigated their diagnostic potential along with CA 19‐9. Interestingly, diagnostic prediction performance of three DAMs—citrulline, GCDC and CMPF— was individually better than CA 19‐9. Citrulline was the only metabolite to reach optimal performance with AUC > 0.8 and was most significant of all (*P* < 0.001). Moreover, a combination of the four DAMs that were common to tissue and serum—BHB, GCDC, CMPF, and citrulline—as well as CA 19‐9 showed the best diagnostic prediction of all possible combinations of two or more features.

Taken together, in this study, the overall metabolome data as well as the metabolites with altered levels between TN and NAT PDACs mainly belonged to pathways related to amino acid metabolism and nucleotide biosynthesis. Both are essential for maintaining cell proliferation and tumor growth, and the amino acid metabolism is a preferred alternative for nutrient acquisition in PDAC [41, 42, 59]. Hence, amino acid metabolism is recently also being explored as a potential therapeutic target [60, 61]. The findings of the present study suggest that residual tumors following neoadjuvant FOLFIRINOX are likely metabolically less active, particularly for pathways related to amino acid and nucleotide metabolism. Moreover, negative correlation of deoxycholate with reduced CA 19‐9 following NAT and positive correlation with survival suggests its potential use for assessment of treatment response. Also, serum levels of citrulline alone or a combination of CA 19‐9 with the four DAMs common to tissue and serum could be used as potential panel of biomarkers to assess response to NAT in PDAC.

The study has certain limitations. First, the proportion of patients with locally advanced disease is higher in the NAT group, which might result in different molecular characteristics of the PDAC in TN and NAT groups. Second, since there is a lack of matched pretreatment tumor samples for the NAT group, samples from TN PDAC patients are used, which weakens the strength of the comparison. Lastly, the presence of prominent intratumor heterogeneity in PDAC may influence the results, as the tissue sample was collected randomly from the tumor bed of each PDAC, while the serum samples likely reflect the global tumor metabolism. Despite these limitations, the study provides further evidence of chemotherapy‐induced suppression of metabolism in PDAC and highlights the need and potential of future investigations in the search for treatment response markers. Future studies aimed at identifying biomarkers for the effect of NAT should be based on larger patient cohorts and comparative metabolomics of serum samples collected at multiple timepoints during neoadjuvant treatment.

## 5 Conclusions

This is the first study to report evidence of using metabolomics to investigate the effects of neoadjuvant chemotherapy in PDAC patients. The residual tumors following neoadjuvant FOLFIRINOX showed suppression of metabolites associated with the amino acid metabolism and nucleotide biosynthetic pathway. Reduced serum CA 19‐9 following NAT correlated negatively with serum deoxycholate and positively with nine amino acid metabolites in NAT tumors. Elevated bile acids and reduced ketone bodies, citrulline and furan fatty acid were identified in both tissue and serum in the NAT group. A serum metabolite panel consisting of deoxycholate, GCDC, BHB, citrulline, and CMPF as biomarkers could potentially be used for early monitoring of treatment response in PDAC patients.

## Conflict of interest

The authors declare no conflict of interest.

## Author contributions

MA designed the study, performed data analysis, and wrote the first draft of manuscript. SJTG performed LC–MS and metabolite identification. AVF performed sample collection and handling. KJL collected clinical data. LE, HR, and KBPE interpreted metabolomics data. IPG contributed to study design and funding acquisition. CSV performed histopathological assessment, funding acquisition, and supervision. All authors contributed to the critical revision of the manuscript and approved the final manuscript.

## Supporting information


**Fig. S1.** Representative histology images of PDAC specimens.
**Fig. S2.** Kaplan–Meier survival curve.
**Fig. S3.** Total ion chromatograms of pooled quality control samples.
**Fig. S4.** Scatter plots showing correlation between metabolite abundance and clinical parameters.
**Fig. S5.** Scatter plots showing correlation between metabolite abundance and % change in serum CA 19‐9 levels following neoadjuvant treatment.

**Table S1.** Clinical characteristics of the study population.
**Table S2.** Raw data for DAMs.
**Table S3.** Correlation analysis of unique DAMs detected both in tissue and serum samples.
**Table S4.** List of key pathways associated with pancreatic tumor metabolome identified.

## Data Availability

Data used in the present study are available from the corresponding author on reasonable request.
