# Supplementary material for: Global metabolomic profiling of tumor tissue and paired serum samples to identify biomarkers for response to neoadjuvant FOLFIRINOX treatment of human pancreatic cancer
Source: Mol Oncol. 2024 Nov 15;19(2):391–411. doi: 10.1002/1878-0261.13759 (PMC11793008; doi:10.1002/1878-0261.13759)
Supplement: Supplementary file 1 — Fig. S1. Representative histology images of PDAC specimens. Fig. S2. Kaplan–Meier survival curve. Fig. S3. Total ion chromatograms of pooled quality control samples. Fig. S4. Scatter plots showing correlation between metabolite abundance and clinical parameters. Fig. S5. Scatter plots showing correlation between metabolite abundance and % change in serum CA 19‐9 levels following neoadjuvant treatment. [file MOL2-19-391-s001.pdf]

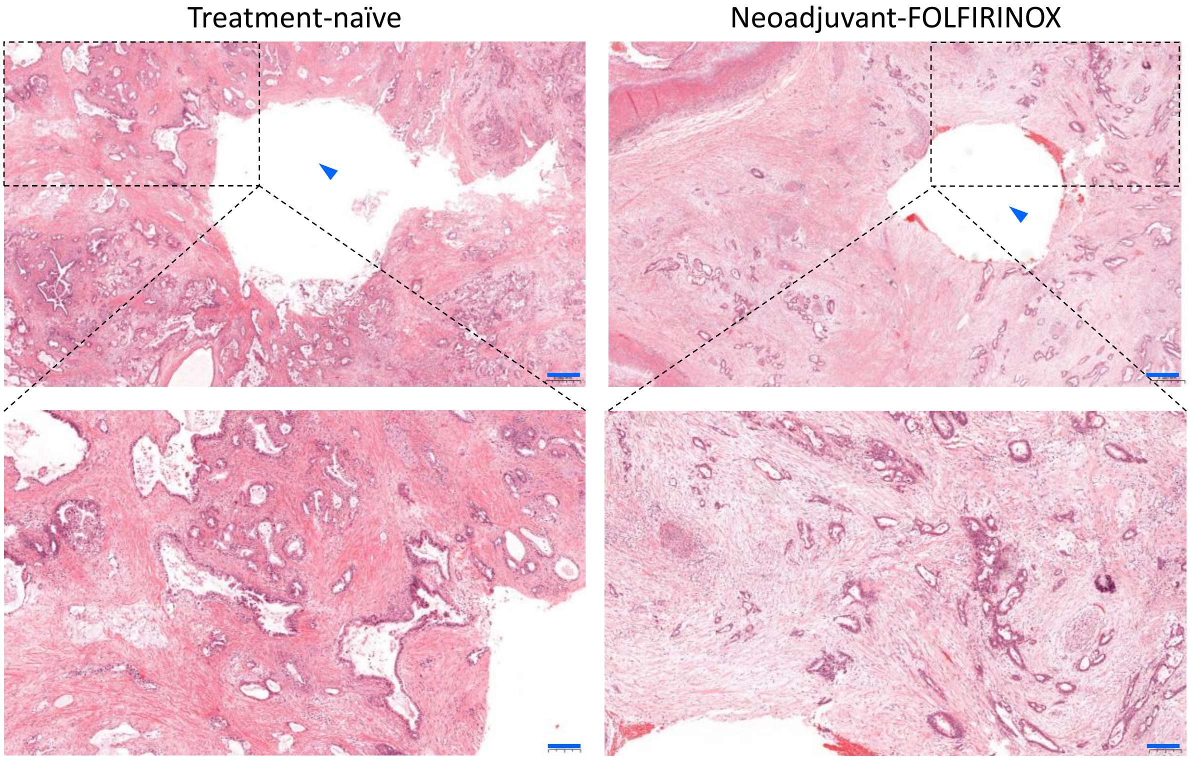

Fig. S1. Representative histology images from treatment-naïve (TN) and neoadjuvantly treated (NAT) PDAC tumor specimens at two different magnifications (upper panel: 2.3x, lower panel: 5.3x). Blue arrowheads indicate the area from which tissue was sampled for metabolomics analysis. Scale bar = 200 µm.

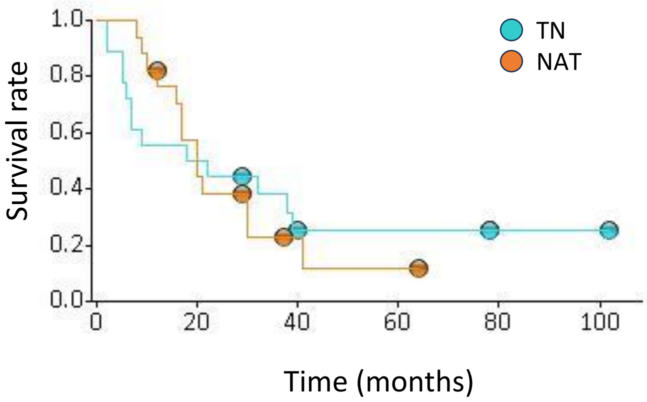

Fig. S2. Kaplan-Meier survival curve. The length of survival calculated from the date of diagnosis. TN, treatment-naïve; NAT, neoadjuvantly treated.

Tissue POS

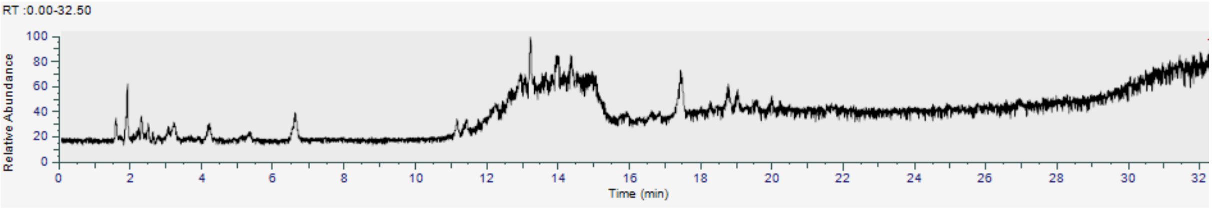

Tissue NEG

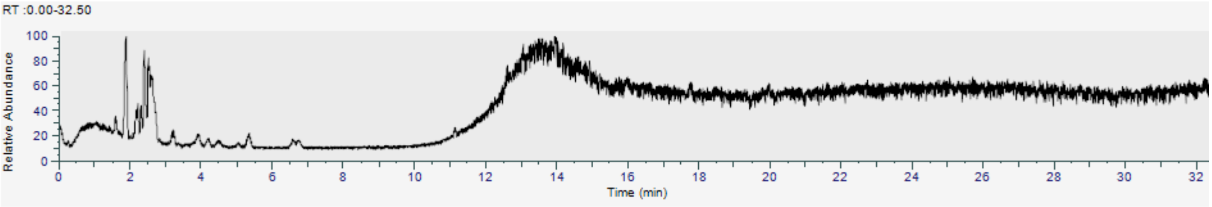

Serum POS

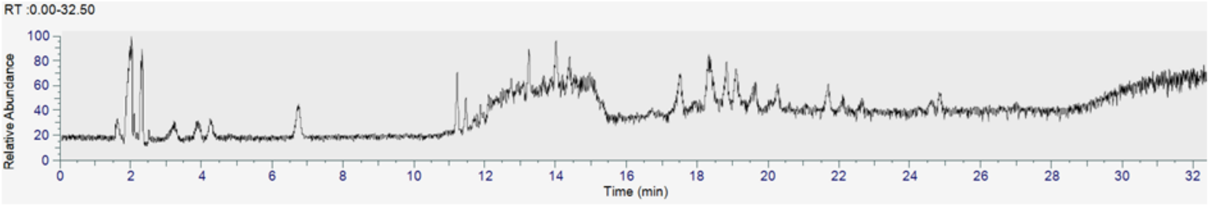

Serum NEG

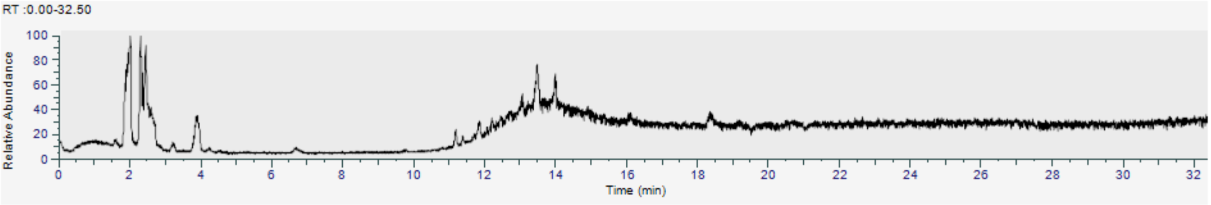

Fig. S3. Total ion chromatograms (TIC) of pooled quality control (PQC) samples for tissue and serum in both positive (POS) and negative (NEG) ionization mode. RT, retention time.

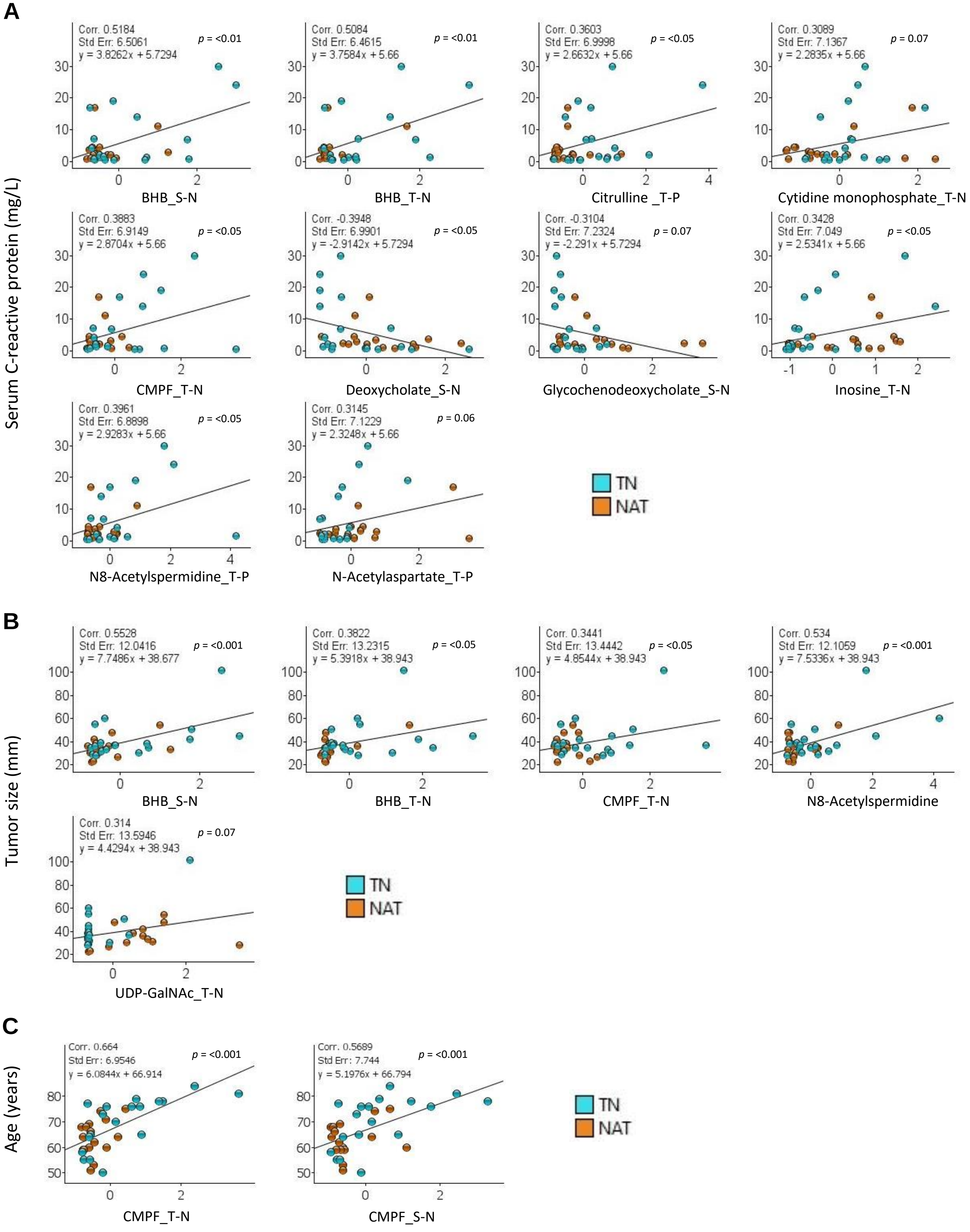

Fig. S4. Scatter plots showing correlation between metabolite abundance and clinical parameters **A.** C-reactive protein, **B.** Tumor size, and **C.** Age. BHB, 3-hydoxybutyric acid; CMPF, 3-carboxy-4-methyl-5-propyl-2-furanpropanoic acid; UDP-GalNAc, uridine-diphosphate-N-acetylgalactosamine; TN, treatment-naïve; NAT, neoadjuvantly treated; S-N, serum negative ion; T-P/T-N, tissue positive/negative ion.

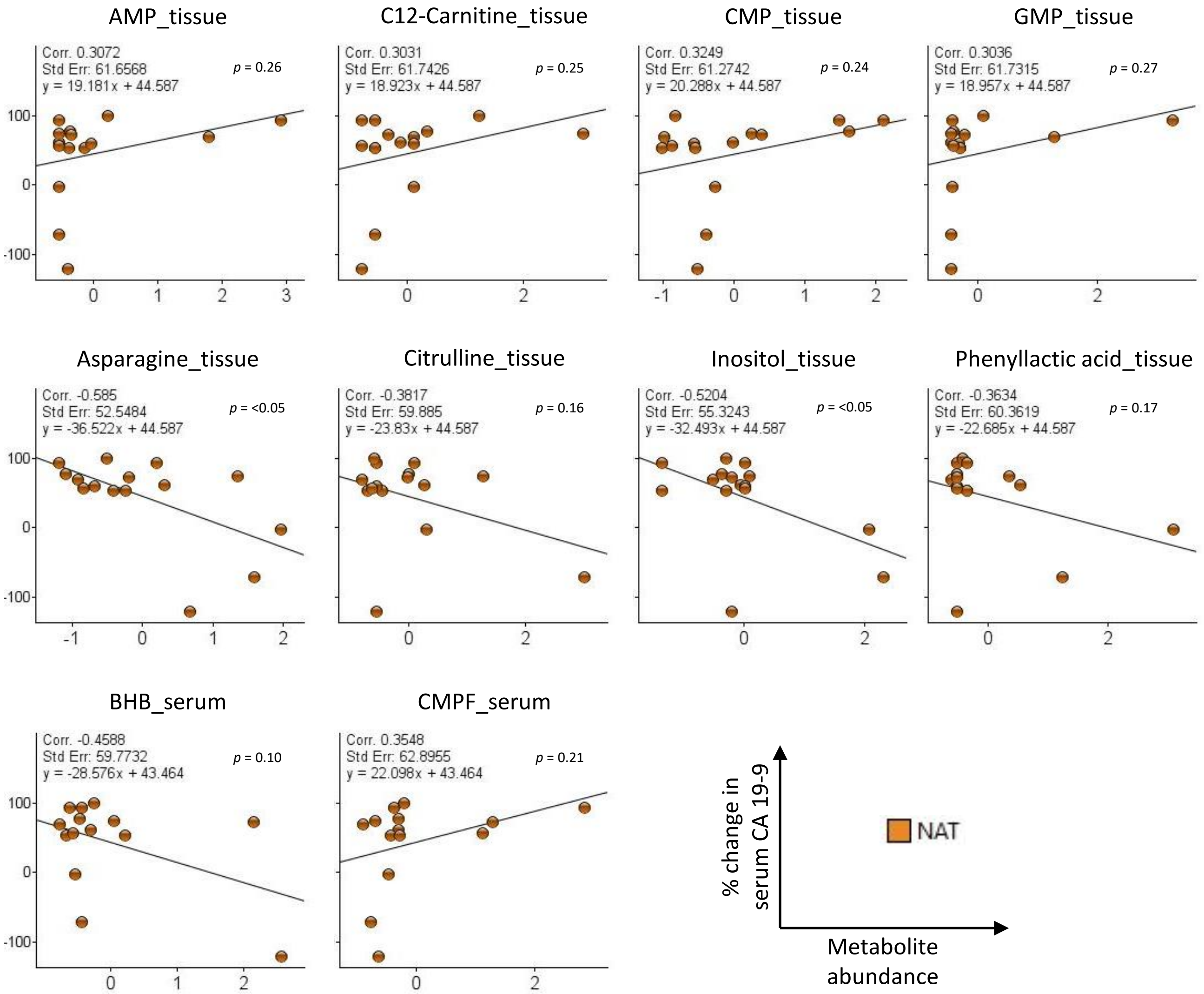

Fig. S5. Scatter plots showing correlation between metabolite abundance and % change in serum CA 19-9 levels following neoadjuvant treatment (NAT samples). AMP, adenosine monophosphate; CMP, cytidine monophosphate; GMP, guanosine monophosphate; BHB, 3-hydoxybutyric acid; CMPF, 3-carboxy-4-methyl-5-propyl-2-furanpropanoic acid; NAT, neoadjuvantly treated.
