# Supplementary material for: Global metabolomic profiling of tumor tissue and paired serum samples to identify biomarkers for response to neoadjuvant FOLFIRINOX treatment of human pancreatic cancer
Source: Mol Oncol. 2024 Nov 15;19(2):391–411. doi: 10.1002/1878-0261.13759 (PMC11793008; doi:10.1002/1878-0261.13759)
Supplement: Supplementary file 2 — Table S1. Clinical characteristics of the study population. Table S2. Raw data for DAMs. Table S3. Correlation analysis of unique DAMs detected both in tissue and serum samples. Table S4. List of key pathways associated with pancreatic tumor metabolome identified. [file MOL2-19-391-s002.zip › mol213759-sup-0002-TablesS1,S3,S4.pdf]

Table S1. Clinical characteristics of the study population.

PC ID	Group	Inclusion year	Gender (0=F, 1=M)	Age (Years)	BMI	Type of co-morbidity	Disease stage (resectable)	Specimen type	NAT regimen (FOLFIRINOX)	CA 19-9 (pre-NAT; U/mL)	CA 19-9 (post-op; U/mL)
20	TN	2015	1	50	23,8	-	PRPC	PPPDE	-	-	677
21	TN	2015	1	77	21,7	1+5	PRPC	PDE	-	-	<5
22	TN	2015	0	65	21,5	2+5	PRPC	DPE	-	-	110
31	TN	2017	1	79	22,5	1+2	PRPC	PDE	-	-	1676
34	TN	2017	1	64	26,4	2	PRPC	PPPDE	-	-	299
38	TN	2017	1	70	23,8	2	PRPC	PPPDE	-	-	<5
40	TN	2017	1	76	20,7	-	PRPC	PPPDE	-	-	35
51	TN	2017	1	58	32,0	1+3+5	PRPC	PDE + VMS	-	-	191
53	TN	2017	0	76	39,6	1+3	PRPC	PDE	-	-	282
72	TN	2018	1	55	25,6	1+3	PRPC	PPPDE	-	-	2932
76	TN	2018	1	55	29,3	-	PRPC	PDE	-	-	230
80	TN	2018	0	73	21,8	1+2+3	PRPC	PPPDE	-	-	11371
90	TN	2020	0	78	34,0	3+5	PRPC	DPE	-	-	178
91	TN	2020	1	65	26,6	1	PRPC	PPPDE	-	-	774
101	TN	2021	1	78	23,4	4+5	PRPC	PPPDE	-	-	493
103	TN	2021	0	76	23,0	2+5	PRPC	PDE	-	-	616
110	TN	2021	1	84	23,3	1+2	PRPC	DPE	-	-	11
111	TN	2021	1	81	26,9	2	PRPC	DPE	-	-	70
35	NAT	2016	1	68	17,3	-	BRPC	PDE+VMS	x4	97	30
39	NAT	2016	1	64	25,2	-	BRPC	PDE	x4	-	93
42	NAT	2017	0	60	21,7	-	PRPC	PDE	x4	878	56
43	NAT	2017	1	60	34,8	-	LAPC	TPE + AMS	x7	272	107
52	NAT	2017	1	53	22,4	-	PRPC	PDE	x4	56	13
61	NAT	2017	0	59	22,9	5	PRPC	PDE	x2	8827	19
66	NAT	2018	0	69	19,1	2	BRPC	PDE + VP	x4	15	7
67	NAT	2018	0	68	25,0	3+5	PRPC	PPPDE	x4	31	53
68	NAT	2018	0	66	27,5	3	BRPC	PPPDE	x5	1035	268
70	NAT	2018	1	71	32,1	2+5	BRPC	PDE	x6	365	145
77	NAT	2018	1	62	29,7	1+3	PRPC	PDE	x4	463	477
81	NAT	2018	0	59	23,5	-	PRPC	PPPDE	x4	768	55
100	NAT	2020	0	51	33,3	-	BRPC	PPPDE	x4	73	34
105	NAT	2020	0	75	24,5	1+4+5	BRPC	PPPDE+VMS	x4	-	31
109	NAT	2021	0	59	21,2	-	BRPC	PDE+VMS	x4	85	187
114	NAT	2021	0	64	28,1	5	BRPC	PPPDE	x4	491	213
118	NAT	2022	1	74	24,7	-	BRPC	PDE	x9	289	80

PC ID	Bilirubin (µmol/L)	Albumin (mg/dL)	CRP (mg/L)	Tumor size (mm)	T stage	N stage	TRG	Survival Dec.2023	Survival (months)	Adjuvant regimen
20	22	45	1,2	35	3	2	-	1	7,9	Folfirinox
21	58	36	7,2	55	3	2	-	0	102,9	FLV
22	5	44	1,3	32	3	0	-	1	18,5	-
31	10	44	<0.6	33	3	2	-	1	2,4	-
34	55	39	4,3	29	3	2	-	1	2,4	-
38	34	41	17	35	3	2	-	0	78,9	-
40	8	44	<0,6	28	3	1	-	0	78,9	Gemcitabine
51	16	43	<0,6	38	3	1	-	1	38,8	GemCap
53	29	39	7	42	3	0	-	1	32,3	NA
72	31	43	0,8	36	2	2	-	1	39,0	GemCap
76	107	41	2,2	39	2	1	-	1	5,8	Folfirinox
80	33	40	1,5	60	3	2	-	1	5,7	-
90	9	45	0,9	51	3	1	-	0	40,7	Gemcitabine
91	272	39	24	45	3	2	-	1	22,9	mFolfirinox
101	38	34	19	37	2	1	-	1	9,9	GemCap
103	341	37	14	30	2	0	-	1	7,0	-
110	5	38	30	102	3	0	-	0	29,8	-
111	6	40	0,6	37	2	2	-	1	6,9	-
35	4	29	0,9	36	3	1	CAP3	1	8,1	FLV
39	7	40	3,5	42	3	1	CAP3	1	41,4	GemCap
42	4	44	0,8	28	3	2	CAP2	1	20,1	GemCap
43	12	44	2,3	35	3	2	CAP3	1	9,0	-
52	6	42	17	38	3	1	CAP2	1	16,8	GemCap
61	4	43	4,4	31	2	2	CAP2	1	21,3	GemCap
66	4	42	3,8	30	3	0	CAP3	1	20,0	Gem
67	9	41	2,4	33	3	2	CAP3	1	17,4	GemCap
68	9	40	2,1	36	2	1	CAP2	1	17,8	Folfirinox
70	6	43	3,3	48	3	2	CAP3	1	30,9	GemCap+FLV
77	24	42	2,2	35	2	1	CAP2	1	30,6	Folfirinox
81	4	42	1	22	2	0	CAP2	0	64,2	Folfirinox
100	3	37	2	48	3	1	CAP2	0	37,1	Folfirinox
105	4	41	1	27	2	2	CAP2	1	12,7	Gemcitabine
109	3	39	3	33	2	1	CAP3	1	10,5	GemCap
114	6	44	4,6	23	2	1	CAP3	0	29,9	Folfirinox
118	5	43	11	54	3	2	CAP2	0	12,9	n/a

Comorbidity type: 1= diabetes mellitus, 2=cardiovascular, 3=hypertension, 4= COPD, 5=other. TN, treatment-naïve; NAT, neoadjuvantly treated; BMI, body-mass index; CA 19-9, Carbohydrate 19-9 antigen; CAP, College of American Pathologists; DP, distal pancreatectomy; PPPD, pylorus-preserving pancreatoduodenectomy; TP, total pancreatectomy; TRG, tumor regression grade; PC, pancreatic cancer; PRPC, primary resectable PC; BRPC, borderline resectable PC; LAPC, locally advanced PC.

Table S3. Correlation analysis of unique DAMs detected both in tissue and serum samples.

**Correlation coefficient (r)**	GCDC (T_N)	GCDC (S_N)	BHB (T_N)	BHB (S_N)	CMPF (T_N)	CMPF (S_N)	Citrulline (T_P)	Citrulline (S_P)
GCDC (T_N)	1	0,36*	0,12	-0,11	-0,24	-0,16	-0,17	-0,21
GCDC (S_N)	0,36*	1	-0,27	-0,35*	-0,42*	-0,36*	-0,02	-0,24
BHB (T_N)	0,12	-0,27	1	0,72**	0,25	0,16	0,54**	0
BHB (S_N)	-0,11	-0,35*	0,72**	1	0,43*	0,38*	0,35*	0,24
CMPF (T_N)	-0,24	-0,42*	0,25	0,43*	1	0,83**	0,1	0,3#
CMPF (S_N)	-0,16	-0,36*	0,16	0,37*	0,83**	1	-0,015	0,41*
Citrulline (T_P)	-0,17	-0,02	0,54**	0,35*	0,1	-0,015	1	0,02
Citrulline (S_P)	-0,21	-0,24	0	0,24	0,3#	0,41*	0,02	1

The significance of correlation between metabolites is indicated with ***p*<0.01 and **p*<0.05 while #*p*<0.1 indicates a trend of difference. BHB, 3-hydoxybutyric acid; CMPF, 3-carboxy-4-methyl-5-propyl-2-furanpropanoic acid; DAMs, differentially abundant metabolites; GCDC, glycochenodeoxycholate; S_N, serum negative; S_P, serum positive; T_N, tissue negative; T_P, tissue positive.

Table S4. List of key pathways associated with pancreatic tumor metabolome identified.

Pathway	# Total	# Hits	Metabolites	*p*-value
Arginine and polyamine biosynthesis	48	16	Acetic acid, AMP, Arginine, Argininosuccinic acid, Aspartate, Citrulline, Fumarate, Glutamine, Glutamate, Ketoglutarate, Maleate, Maleic acid, N-acetylornithine, N-Acetylserine, Ornithine, Phosphoric acid, Pyrophosphoric acid	<0.001
Aromatic amino acid biosynthesis	47	17	Aspirin, p-coumaric acid, 4-coumarate, Glutamine, Glutamate, Indole, Inositol, Ketoglutarate, Mannose-6-Phosphate, N-Acetylserine, Phenylalanine, Phosphoric acid, Pyrophosphoric acid, Pyruvic acid, Ribose-5-phosphate, Ribonolactone, Serine, Tryptophan, Tyrosine	<0.001
Aspartate metabolism	74	19	AMP, Aspartate, Cysteine, Cystathionine, 2,6-Diaminopimelic acid, Glutamine, Glutamate, Ketoglutarate, Lysine, Methionine, Methylmalonate, N-Acetylserine, N-acetylglucosaminolactone, Phosphoric acid, Pyrophosphoric acid, Pyruvic acid, Threonine, Ribose-5-phosphate, Ribonolactone	<0.001
Branched chain amino acid biosynthesis	37	12	Acetoacetate, Adipic acid, Glutamate, 4-hydroxy-2-oxovaleric acid, Hydroxyethyl methacrylate, Isoleucine, Ketoglutarate, N-Acetylserine, 2-Oxobutyric acid, 6-Oxohexanoic acid, Pyruvic acid, Ribonolactone, Threonine, Valine	<0.01
Citrulline metabolism	35	13	AMP, Arginine, Argininosuccinic acid, Aspartate, Citrulline, Fumarate, Glutamine, Glutamate, Hydroxyproline, Ketoglutarate, Maleate, Maleic acid, N-acetylalanine, N-Acetylserine, Ornithine, Phosphoric acid, Pyrophosphoric acid	<0.01
Purine nucleotide salvage	54	13	AMP, Aspartate, Fumarate, Glutamine, Glutamate, Guanosine, GMP, Hypoxanthine, Inosine, IMP, Maleate, Maleic acid, N-Acetylserine, Phosphoric acid, Pyrophosphoric acid, Ribose-5-phosphate	<0.001
Pyrimidine ribonucleosides salvage	29	8	CMP, Glutamine, Glutamate, N-Acetylserine, Phosphoric acid, Pseudouridine, Pyrophosphoric acid, Uridine, UMP	<0.001
Glycolysis and TCA cycle	59	18	AMP, Fructose 1,6-diphosphate, Fumarate, Glucose-6-phosphate, Glucose 1,6-diphosphate, Isocitrate, Ketoglutarate, Malate, Maleic acid, Methylmalonate, 3-Phosphoglycerate, Phosphoric acid, Pyruvic acid, Ribose-5-phosphate, Ribonolactone, trans-Aconitate	<0.01
Sucrose (anaerobic) degradation	41	17	Allulose, AMP, Fructose 1,6-diphosphate, Glucose, Glucose-6-phosphate, Glucose 1,6-diphosphate, Inositol, Lactic acid, Maltose, 3-Phosphoglycerate, Phosphoric acid, Pyrophosphoric Acid, Pyruvic acid, Ribose-5-phosphate, Ribonolactone, Threonic acid, UDP-glucose, UDP-galactose	<0.001
Lysine degradation	97	16	Acetic acid, Acetoacetate, β-Alanine, Aminoadipate, Glutamate, Gly-Leu, 4-hydroxy-2-oxovaleric acid, Ketoglutarate, Lysine, Methylmalonate, N-Acetylserine, 2-Oxobutyric acid, Pyrophosphoric Acid, Pyruvic acid, Ribonolactone, Valine	<0.05

These pathways and related metabolites were identified using the Metabolika module available in the Compound discoverer software. AMP, adenosine monophosphate; CMP, cytidine monophosphate; GMP, guanine monophosphate; IMP, inosine monophosphate; UDP, uridine diphosphate; UMP, uridine monophosphate.
